# Single-cell genome-wide association reveals that a nonsynonymous variant in *ERAP1* confers increased susceptibility to influenza virus

**DOI:** 10.1016/j.xgen.2022.100207

**Published:** 2022-11-09

**Authors:** Benjamin H. Schott, Liuyang Wang, Xinyu Zhu, Alfred T. Harding, Emily R. Ko, Jeffrey S. Bourgeois, Erica J. Washington, Thomas W. Burke, Jack Anderson, Emma Bergstrom, Zoe Gardener, Suzanna Paterson, Richard G. Brennan, Christopher Chiu, Micah T. McClain, Christopher W. Woods, Simon G. Gregory, Nicholas S. Heaton, Dennis C. Ko

**Affiliations:** 1 Department of Molecular Genetics and Microbiology, School of Medicine, Duke University, 0048B CARL Building Box 3053, 213 Research Drive, Durham, NC 27710, USA; 2 Duke University Program in Genetics and Genomics, Duke University, Durham, NC 27710, USA; 3 Center for Applied Genomics and Precision Medicine, Department of Medicine, Duke University, Durham, NC 27710, USA; 4 Hospital Medicine, Division of General Internal Medicine, Department of Medicine, Duke Regional Hospital, Durham, NC 27705, USA; 5 Department of Biochemistry, School of Medicine, Duke University, Durham, NC 27710, USA; 6 Section of Infectious Diseases and Immunity, Imperial College London, London, W12 0NN, UK; 7 Durham Veterans Affairs Health Care System, Durham, NC 27705, USA; 8 Division of Infectious Diseases, Department of Medicine, School of Medicine, Duke University, Durham, NC 27710, USA; 9 Duke Molecular Physiology Institute, Duke University Medical Center, Durham, NC 27710, USA; 10 These authors contributed equally; 11 Lead contact

## Abstract

During pandemics, individuals exhibit differences in risk and clinical outcomes. Here, we developed single-cell high-throughput human *in vitro* susceptibility testing (scHi-HOST), a method for rapidly identifying genetic variants that confer resistance and susceptibility. We applied this method to influenza A virus (IAV), the cause of four pandemics since the start of the 20^th^ century. scHi-HOST leverages single-cell RNA sequencing (scRNA-seq) to simultaneously assign genetic identity to cells in mixed infections of cell lines of European, African, and Asian origin, reveal associated genetic variants for viral burden, and identify expression quantitative trait loci. Integration of scHi-HOST with human challenge and experimental validation demonstrated that a missense variant in *endoplasmic reticulum aminopeptidase 1* (*ERAP1*; rs27895) increased IAV burden in cells and human volunteers. rs27895 exhibits population differentiation, likely contributing to greater permissivity of cells from African populations to IAV. scHi-HOST is a broadly applicable method and resource for decoding infectious-disease genetics.

## In brief

Schott et al. developed scHi-HOST, a GWAS platform leveraging scRNA-seq to assign cell identity and detect SNPs associated with pathogen resistance and gene expression in mixed infections of lymphoblastoid cell lines. scHi-HOST revealed an SNP associated with influenza burden. This association is confirmed in cell culture and human flu challenge.

## Graphical Abstract

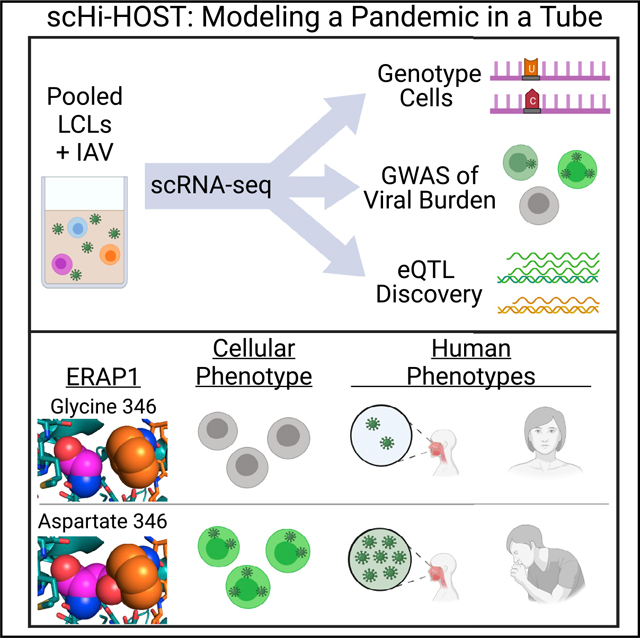

## INTRODUCTION

Infectious diseases have been a leading cause of mortality throughout human history. These past exposures to pathogens have conferred strong selective pressures on the human genome, driving adaptation as resistance alleles become more common. In pursuit of genetic differences that impact resistance and susceptibility, approaches using human populations have focused on the identification of common or rare variants. For influenza A virus (IAV), small, underpowered genome-wide association studies (GWASs) using a common variant approach have failed to identify any genome-wide significant loci.^1–3^ Coupling small candidate association studies with functional evidence has revealed associations of common variants in *IFITM3* with severe influenza.^4,5^ On the other end of the frequency spectrum, rare variants predisposed to severe influenza have been identified in *IRF7*^6^ and *TLR3*.^7^ Complementary *in vitro* approaches for identifying human genetic variation can control for differences in exposure, pathogen genetic diversity, co-morbidities, and access to medical care. Such approaches could be used to broadly probe human genetic diversity for resistance and susceptibility to emerging pathogens and would be a powerful tool, especially as outbreaks develop in single geographic locations.

Previously, we developed a cellular GWAS platform, high-throughput human *in vitro* susceptibility testing (Hi-HOST), in which lymphoblastoid cell lines (LCLs; Epstein-Barr virus (EBV) immortalized B cells) from hundreds of genotyped individuals are exposed to identical doses of a pathogen and assessed for cellular host-pathogen traits including entry,^8,9^ replication,^10^ cell death,^11–13^ and cytokine response.^10,14,15^ This approach revealed human genetic differences associated with cellular traits that are also associated with infectious disease risk^8,16,17^ and outcomes.^11,18^ However, Hi-HOST and similar studies are labor and time intensive and can be skewed due to batch effects in measuring hundreds of individual cellular infections over a period of months or even years. Additionally, incorporation of new technology for simultaneous identification of expression quantitative trait loci (eQTLs) combined with GWASs of pathogen measurements could help fill the mechanistic gap from genetic variant to cellular infection phenotype.

Here, we present a rapid, high-throughput approach called single-cell Hi-HOST (scHi-HOST) that uses single-cell RNA sequencing (scRNA-seq) for simultaneous identification of alleles associated with both gene expression changes and genetic resistance to IAV. This method leverages the genetic diversity of dozens of individuals from multiple populations in a single infection and reveals both phenotypic differences in IAV susceptibility among populations and a common missense variant in *ERAP1* (rs27895) associated with IAV burden in LCLs and a human flu challenge study. Coupling scHi-HOST with human challenge studies is a broadly generalizable approach for understanding human genetic susceptibility to infectious disease using a small number of individuals in a rapid time frame.

## RESULTS

### A single-cell approach for discovering genetic resistance to infection

**Figure 1.**
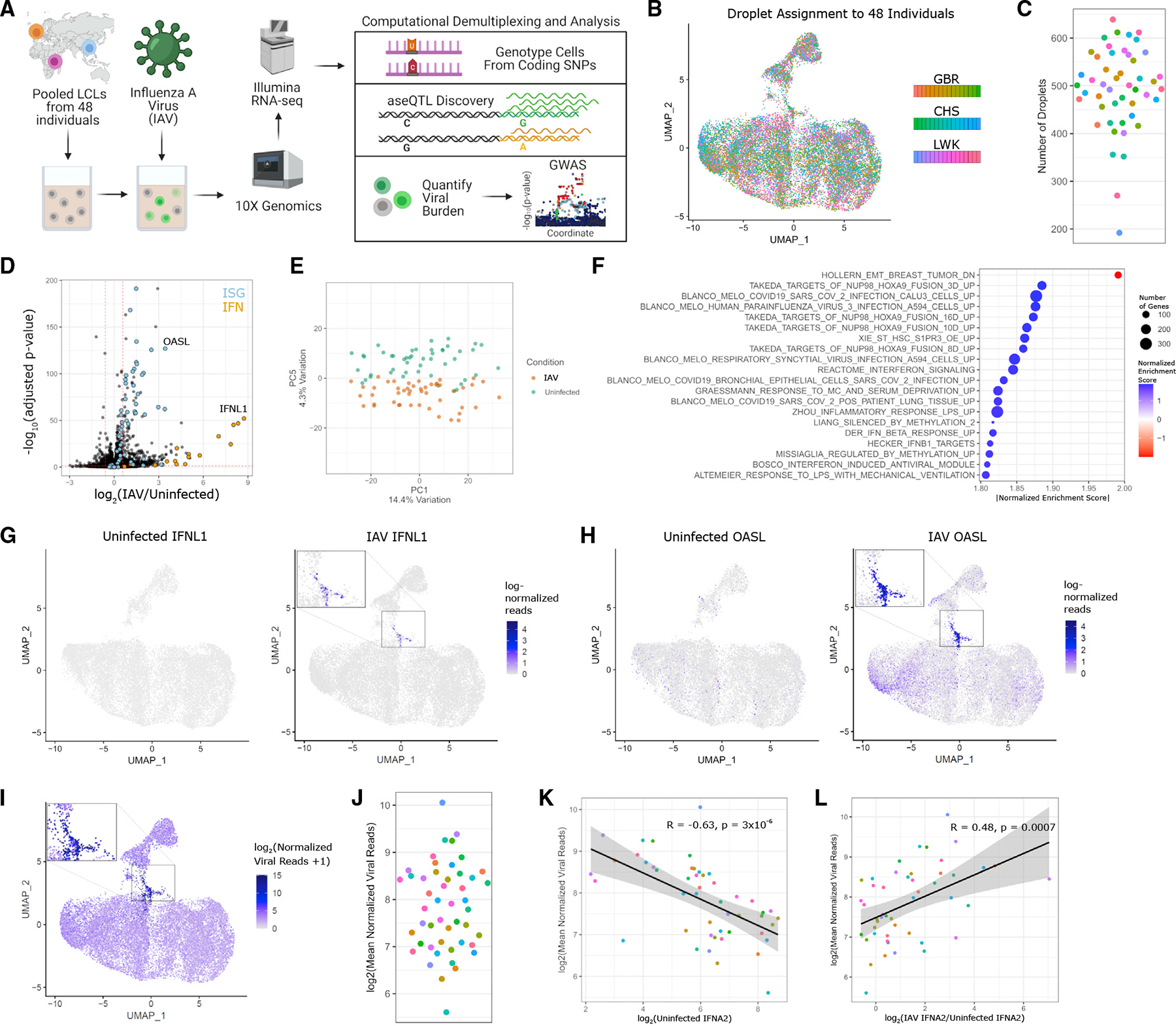
scHi-HOST is a rapid platform for cellular host-pathogen genome-wide association (A) Flowchart of scHi-HOST with influenza A virus. (B) UMAP plot demonstrates assignment of individual cells to one of 48 genotyped LCLs for uninfected and influenza A virus (IAV)-infected scHi-HOST samples. Color coded by shades for each population. (C) The number of individual cells measured for each LCL in the scRNA-seq analysis. Color coding is the same as (B). (D) Volcano plot of pseudo-bulk analysis of uninfected versus infected LCLs revealed upregulation of interferons (IFNs) and ISGs. Pseudo-bulk analysis was performed by aggregating all cells of the same LCL identity (total of 48 pseudo-bulk samples) and performing differential expression in DESeq2.^24^ Genes are color coded by functional class: orange, Browne IFN responsive genes (GSEA C2 geneset); blue, all 21 human IFN genes; black, all other genes. Dotted red horizontal line indicates p = 0.05. Dotted red vertical lines indicate fold change >1.5 or <0.5 relative to uninfected. (E) Pseudo-bulk analysis shows uninfected transcriptomes and infected transcriptomes segregate along PC5. (F) GSEA shows upregulation of ISGs and other viral response gene sets. Plotted gene sets are top 20 absolute normalized enrichment score, all FWER p < 0.05. (G) UMAP plots of *IFNL1* in uninfected and IAV-infected LCLs. (H) UMAP plots of *OASL* in uninfected and IAV-infected LCLs. (I) UMAP plot demonstrates highly variable IAV burden across individual cells and a cluster of highly infected cells. UMAP plots were generated by combining uninfected and IAV samples for normalization and plotting each condition separately with Seurat.^24^ Viral reads were detected in 23,346 out of 23,684 cells (median = 7.9 reads, with a range of 0.4–33,960; summing to a total count of 6.4 million normalized viral reads). In contrast, uninfected cells had a total of 19.6 normalized viral reads, with reads detected in only 23 out of 20,129 cells. These likely represent index-hopped artifacts and not true contaminants. (J) Mean viral reads across 48 LCLs. Phenotype was generated by normalizing by total read depth per cell (human and viral reads) and aggregating all cells of the same LCL identity. Color coding is the same as (B). (K) Negative correlation of baseline *IFNA2* and viral burden. *IFNA2* was chosen as it has the highest baseline expression, though similar results were observed with other expressed IFNα genes (Figure S1C). Correlation coefficient and p value from Spearman’s correlation. (L) Positive correlation of induced *IFNA2* and viral burden. Positive correlation between induction of gene expression and viral burden was similarly observed for other IFNα genes (Figure S1D). Correlation coefficient and p value from Spearman’s correlation

We developed scHi-HOST to integrate the identification of common human genetic variants that impact gene expression and cellular infection traits into a single assay. scHi-HOST uses scRNA-seq for simultaneous genotypic assignment of thousands of individual cells and dense phenotyping of host and virus. The scHi-HOST pipeline has 4 steps: (1) infection and scRNA-seq data generation, (2) assignment of individual cells to genotyped individuals, (3) GWAS of pathogen-based burden phenotypes, and (4) eQTL identification of host gene expression (Figure 1A). In one experiment, an equal number of LCLs from 48 individuals across 3 populations (Luhya in Webuye, Kenya [LWK], British in England and Scotland [GBR), and Southern Han Chinese [CHS] from the 1000 Genomes Project^19^) were mixed and infected for 24 h with a laboratory adapted human H1N1 IAV strain (A/Puerto Rico/8/34, PR8). This experiment is referred to as scHH-LGC, based on the populations included. Consistent with previous reports of B cells being direct targets for IAV entry,^20–22^ LCLs were highly infectable by IAV. A scRNA-seq library was prepared using 10x Genomics technology,^23^ targeting a recovery of ~10,000 droplets/well and performing next-generation sequencing at ~100,000 reads/droplet. Mixed LCLs were frozen into aliquots that can be thawed and screened for future pathogens or other stimuli in <1 week.

Following next-generation sequencing, SNPs in the oligo-dT-primed cDNA reads facilitated the unequivocal assignment of nearly all individual cells to one of the 48 genotyped LCLs using Demuxlet^25^ (Figures 1B; median number of individual cells for each LCL = 501, Figure 1C). We achieved a singlet rate of 73% with 99.9% of singlets assigned to a single genotype with >99% confidence.

LCLs displayed a transcriptional response similar to other IAV-infected cell types.^26,27^ Differentially expressed genes (DEGs) were dominated by upregulation of interferon (IFN) and IFN-stimulated genes (ISGs) (Figure 1D; Table S1). Principal-component analysis (PCA) showed convincing separation of uninfected and IAV-infected LCLs along PC5, accounting for 4.3% of the variation (Figure 1E). Gene set enrichment analysis (GSEA)^28^ confirmed upregulation of ISGs along with multiple other gene sets for viral infection including multiple respiratory virus response gene sets, IFN-induced antiviral modules, and the inflammatory response (Figure 1F, all family-wise error rate [FWER] p < 0.05; Table S2).

We found that induction of IFN is widely variable and only detected in a minority of cells (Figure 1G). In contrast, ISGs, including *OASL*, while variably expressed, are more broadly induced throughout the infected culture (Figure 1H). Thus, while the antiviral response is robust, it is driven by a small number of cells that highly induce IFN to then induce ISGs more broadly.

To determine if this transcriptional heterogeneity can be explained by cell-to-cell differences in viral burden, we mapped and quantified viral transcripts, given that poly-adenylated IAV RNA was captured with the LCL transcriptome. A uniform manifold approximation and projection (UMAP) plot revealed that most cells have low, but detectable, levels of viral reads, but there is a cluster of high-burden cells with a distinct host transcriptional response (Figure 1I). This high level of heterogeneity is consistent with previous work using scRNA-seq in IAV-infected A549 lung epithelial cells.^29^ We observed the most highly burdened cells in the culture expressed the highest levels of IFNs and ISGs in response to virus. Aggregating the IAV reads for each of the 48 LCLs revealed large differences, with the mean influenza reads varying ~20-fold across different LCLs (Figure 1J). It was noted that viral burden did not correlate with the copy number of Epstein-Barr virus (EBV) (Figure S1A), used by the 1000 Genomes Project in immortalizing the LCLs, or with the number of each LCL recovered in the scHi-HOST experiment (Figure S1B). Thus, there is wide variation in viral burden across cells at the single-cell level and in aggregate across LCLs, and these differences correlate with differences in the expression of important antiviral genes.

Comparison of gene expression in uninfected and infected LCLs versus viral burden revealed that high IFNα expression at baseline in the uninfected state is correlated with low viral burden in infected cells at 24 h (Figures 1K and S1C). In contrast, after infection, high induction of IFNα is correlated with high viral burden (Figures 1L and S1D). Thus, high baseline IFNα levels prior to infection appear to be protective; however, during infection, high levels of IAV replication lead to the highest levels of IFNα induction in individual cells. This is consistent with a previous study using scRNA-seq of IAV-infected cells to link basal expression to susceptibility to infection.^30^

In summary, in a single infection, we deconvoluted cells from 48 different individuals and observed highly variable flu burden and transcriptional phenotypes. These methods revealed that baseline IFN levels contribute to resistance against influenza infection. We next turned to identifying human genetic differences that regulate cellular response to infection.

### Common human genetic differences impact transcriptional response to IAV in cells

**Figure 2.**
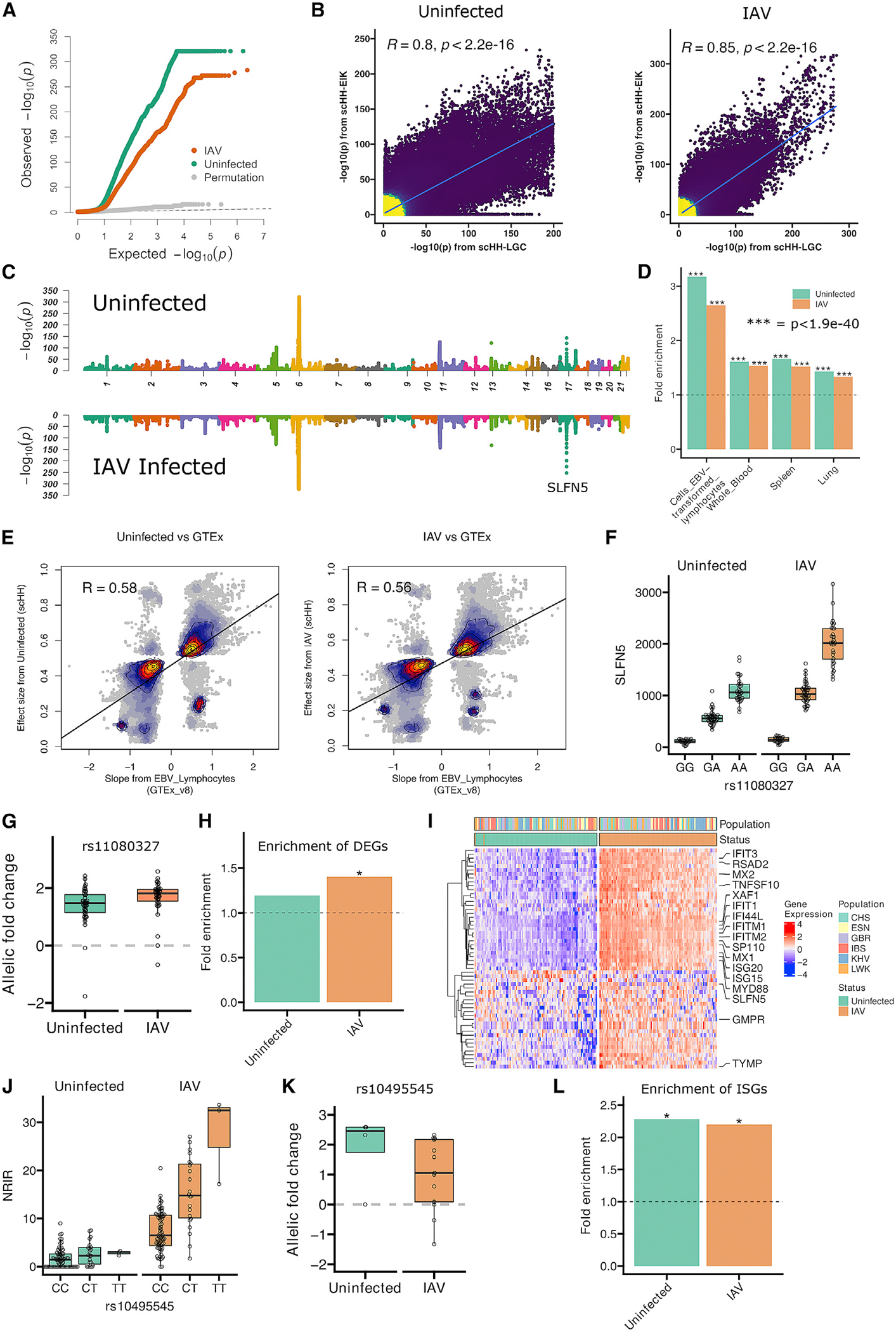
Common human genetic differences regulate transcriptional response during cellular influenza infection (A) QQ plot of observed, permutated, and expected −log(p values) for eQTLs demonstrates deviation of observed p values generated using RASQUAL from neutral expectation for both uninfected and IAV-infected LCLs. (B) Correlation of eQTLs identified by scHH-LGC and scHH-EIK. −log p values for all SNP-gene pairs with p <0.05 in scHH-LGC were plotted against −log p for the same SNP-gene pairs in scHH-EIK. Correlation coefficient and p values are from Pearson correlation. (C) Miami plot of *cis*-eQTLs for uninfected and IAV-infected cells identified in the combined scHH-LGC + scHH-EIK dataset in 1-MB windows using FDR-corrected p values. (D) scHi-HOST eGenes from uninfected and IAV-infected LCLs were highly enriched in GTEx v.8. scHi-HOST eGenes were defined using a 2-step FDR <0.05, and Fisher exact test was used to calculate the significance of enrichment of scHi-HOST eGenes against GTEx eGenes (FDR < 0.01). (E) Comparison of effect sizes from scHi-HOST eQTLs against GTEx eQTLs from EBV lymphocytes. For scHi-HOST eQTLs at FDR <0.05, 9.5% of uninfected eQTLs (from 1,090 out of 2,265 eGenes) and 7.6% of IAV-infected eQTLs (from 1,266 out of 3,326 eGenes) are significant eQTLs in GTEx LCLs. The effect sizes of scHi-HOST eQTLs generated by RASQUAL (y axis) are expressed as the ratio of expression of the alternative allele/(alternative + reference allele). The effect sizes of GTEx eQTLs (x axis) are expressed as normalized effect size, defined as the slope of the linear regression line with the alternative allele as the effect allele. Color and contour lines depict the density of points. Scatterplot of eQTL effect sizes from scHi-HOST uninfected or IAV-infected against GTEx EBV lymphocytes suggest a high correlation of 0.58 or 0.56 from Pearson correlation, respectively. (F and G) rs11080327, the most strongly associated *cis*-eQTL outside of the MHC region (uninfected 2-step FDR = 5.3 × 10^−140^, IAV 2-step FDR = 1.3 × 10^−249^), is associated with expression of *SLFN5* based on linear regression of genotypic medians (F) and allelic imbalance for uninfected and infected LCLs (G). As *SLFN5* is an ISG, the expression of the gene is higher in IAV-infected LCLs, and the association in that context is stronger. (H) IAV-induced differentially expressed genes (DEGs; p < 0.05, fold change > 1.5) are significantly enriched in IAV-infected eGenes. DEGs between uninfected and IAV infected were identified using a pseudo-bulk approach as described in the STAR Methods. Fisher’s exact tests were used to test the significance of overlap between DEGs and eGenes and to calculate p values. Strong enrichment is observed only with IAV infection (*p = 6.8 × 10^−3^). (I) Heatmap of expression of the 56 DEGs (p < 0.05; absolute fold change [FC] > 1.5) that are also eGenes. Numerous ISGs are labeled. LCLs are color coded by population. (J and K) Genotypic median plots (J) and allelic imbalance plots (K) for the association of rs10495545 with *NRIR* (2-step FDR = 0.0003 for IAV infected, 2-step FDR = 1.0 for uninfected). *NRIR* is induced 4.95-fold with IAV infection and is an IAV-specific eQTL. (L) Enrichment analysis of ISGs in eGenes from uninfected and IAV-infected LCLs. Strong enrichment is observed under both conditions by Fisher’s exact test, consistent with LCLs having baseline IFN production in the uninfected state (*p < 0.001)

We identified human SNPs associated with variation in gene expression in uninfected and infected cells using RASQUAL,^31^ with pseudo-bulk gene expression aggregated for each LCL. RASQUAL tests for the association of SNPs versus gene-expression level by measuring both linear regression among genotypes and allele-specific expression in heterozygous individuals. Observed −log(p values) deviated strongly from theoretical and empirical null distributions (using RASQUAL’s permutation function; see STAR Methods), indicating many true positives under both conditions (Figure 2A). We compared the results of RASQUAL analysis on scHH-LGC with a second scHi-HOST experiment of 48 additional LCLs from Esan from Nigera (ESN), Iberian from Spain (IBS), and Kinh from Vietnam (KHV) (referred to as scHH-EIK) and observed a strong correlation between the two datasets (Figure 2B). An analysis of design considerations for scHi-HOST is provided in the STAR Methods (Figure S2).

The scHH-LGC and scHH-EIK datasets were combined to provide the greatest power. The most significant eQTLs were detected in both uninfected and IAV-infected conditions (Figure 2C). A 2-step approach was used to identify eGenes (unique genes associated with eQTLs) utilizing Benjamini-Hochberg correction for both the SNPs tested in each window and for the number of genes tested. For uninfected and IAV-infected LCLs at a 2-step false discovery rate (FDR) <0.05, we detected 2,265 and 3,326 eGenes, respectively (Table S3). Many of the eQTL associations replicated in the GTEx dataset.^32^ We observed 3.17-fold enrichment (p = 1.26 × 10^−117^) for uninfected eGenes in GTEx LCLs (GTEx FDR < 0.01) and 2.65-fold enrichment (p = 6.02 × 10^−140^) for IAV eGenes, representing 33.01% of the eGenes from the infected sample (Figure 2D). Further, enrichment was observed across multiple tissues in GTEx, including whole blood, spleen, and lung (Figure 2D; all p < 1.9 × 10^−40^). We also observed high correlation of effect size between scHi-HOST eQTLs and GTEx eQTLs (Figure 2E; p < 2.16 × 10^−16^). Thus, as others have noted,^33^ LCLs serve as a valid system for identification of eQTLs that are relevant in many tissues, and we have determined that eQTLs identified in LCLs with scHi-HOST are reproducible across other LCL datasets, related primary tissues, and even other tissues.

The most significant association outside of the human leukocyte antigen (HLA) region in both uninfected and infected LCLs was the association of rs11080327 with *SLFN5* (Figures 2F and 2G; uninfected 2-step FDR = 5.3 × 10^−140^, IAV 2-step FDR = 1.3 × 10^−249^). Notably, the expression of this gene increased with IAV infection (1.80-fold; adjusted p = 0.004) (consistent with *SLFN5* being an ISG^34^), and the effect size of the SNP (allelic fold change^35^) was greater in the infected LCLs (Figure 2G; uninfected allelic fold change [aFC] = 2.6; IAV-infected aFC = 3.6; p = 0.0001).

We observed an enrichment of IAV-induced genes (see Figure 1D) in the IAV eGenes (1.4-fold enrichment; p = 6.8 × 10^−3^) but not in the uninfected eGenes (1.19-fold enrichment; p = 0.18), indicating that context-specific eQTLs can be uncovered by the scHi-HOST approach (Figure 2H). In total, we detected 56 DEGs that were also eGenes, many of which were also ISGs (Figure 2I). For example, we discovered an eQTL (rs10495545) for the long noncoding (lncRNA) *negative regulator of IFN response* (*NRIR*),^36^ also known as AC017076.5, and lncRNA-CMPK2). This lncRNA is induced 4.95-fold during IAV infection in LCLs and is an eQTL only in the IAV-infected state (Figures 2J and 2K; p = 0.0003 for IAV infected, p = 1.0 for uninfected). ISGs are enriched in both uninfected and IAV-infected scHi-HOST datasets (Figure 2L), consistent with LCLs having baseline expression of ISGs that is induced further during IAV infection.

### Integration of scHi-HOST and functional annotation reveals a nonsynonymous SNP in *ERAP1* that increases IAV susceptibility

While scHi-HOST is a powerful method for eQTL identification in response to pathogens, it also serves as a method for rapid association testing of functional subsets of SNPs. We tested for genome-wide association using the linear mixed-model method implemented in EMMAX,^37^ using a kinship matrix to control for relatedness and sex as a covariate. Given the modest size of our combined scHi-HOST dataset (n = 96), even associations that surpass genome-wide significance (traditionally p<5 × 10^−8^) must be evaluated carefully for corroborating evidence. For the phenotype of mean IAV burden, a single SNP (rs1923314; p = 1.8 × 10^−8^) surpassed genome-wide significance (Figures S3A and S3B; full summary statistics available at the Duke Research Data Repository, https://doi.org/10.7924/r4g163n0s.). However, this SNP is in a gene desert and is not a *cis*-eQTL, making functional follow up challenging. Therefore, we examined statistical evidence of association for SNPs that have functional consequences: *cis*-eQTLs as identified above and nonsynonymous SNPs based on annotation.

**Figure 3.**
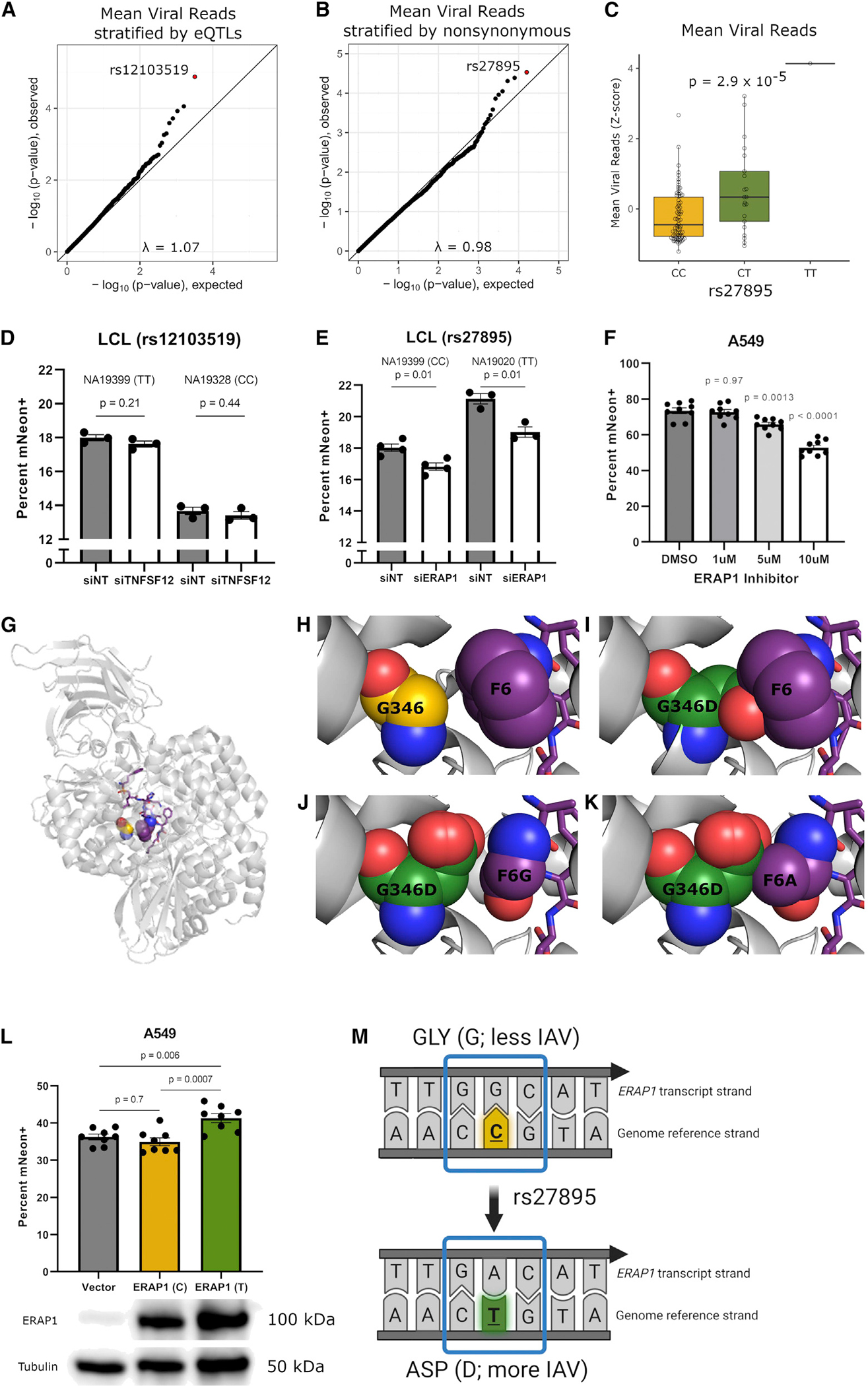
A nonsynonymous variant in *ERAP1* regulates IAV burden in cells (A) Stratified QQ plot for association with mean IAV reads restricted to RASQUAL 2-step FDR eQTLs (FDR < 0.05; MAF > 0.1). Plotting eQTLs reveals an SNP associated with expression of *TNFSF12* that has a lower p value than expected by chance. (B) Stratified QQ plot for association with mean IAV reads restricted to nonsynonymous SNPs. Plotting nonsynonymous variants (MAF >0.1) reveals an SNP in *ERAP1* that has a p value lower than expected by chance. (C) Genotype median plot for mean IAV reads as a function of rs27895 genotype. P value from EMMAX and corrected for genomic inflation factor. (D) RNAi against *TNFSF12* in either NA19399 (TT for rs12103519) or NA19328 (CC for rs12103519) demonstrates reducing *TNFSF12* expression (mean 87% knockdown ±4% [SEM] for NA19328 and mean 73% knockdown ±6% [SEM] for NA19399) does not significantly affect the percentage of IAV-infected cells at 24 h in either LCL. Replicates from 3 separate experiments were normalized to correct for between-experiment variation. p values are from unpaired t tests. (E) RNAi against *ERAP1* in either NA19020 (TT for rs27895) or NA19399 (CC for rs27895) demonstrate that reducing *ERAP1* expression (mean 80% knockdown ±14% [SEM] for NA19020 and mean 70% knockdown ±8% [SEM] for NA19399) decreases the percentage of IAV-infected cells at 24 h in both LCLs. Replicates from 4 separate experiments were normalized to correct for between-experiment variation. One experiment involving NA19020 was excluded because no knockdown was detected. p values are from unpaired t tests. (F) Specific ERAP1 inhibitor, ERAP1-IN-1 (CAS: 865273-97-8, MedChemExpress) demonstrates dose-dependent reduction in the percentage of IAV-infected cells at 24 h in A549s. Replicates from 3 separate experiments were normalized to correct for between-experiment variation. p values are from ordinary one-way ANOVA with Dunnett’s multiple comparisons test using DMSO (vehicle) control. (G) Interaction between ERAP1 residue 346 and an ERAP1 peptide inhibitor. Ribbon diagram of the crystal structure of ERAP1 (light gray, cartoon) bound to a 10-mer peptide inhibitor (purple) (PDB: 6RQX). The carbon atoms of ERAP1 residue 346 and the sixth position of the peptide are shown as orange-, yellow- and purple-colored spheres, respectively. (H) Wild-type ERAP1 G346 and F6 of the 10-mer peptide inhibitor have multiple favorable side chain-backbone van der Waals contacts. (I) However, steric clash between the ERAP1 side chain and the 10-mer peptide inhibitor occurs when G346 is mutated to an aspartate (carbon atoms colored green) using *in silico* mutagenesis. (J) Rare rotamers of ERAP1 G346D do not clash with a peptide in which F6 is replace by a glycine but provide no favorable contacts. (K) Even the addition of only a β carbon at position 6 of the 10-mer peptide inhibitor, i.e., an alanine residue, clashes with ERAP1 (G346D). (L) Overexpression of alternative alleles of *ERAP1* in A549 cells demonstrates that the T allele of rs27895 (aspartate) increases the percentage of IAV-infected cells at 24 h. 8 biological replicates from 3 separate experiments were normalized to correct for between-experiment variation. p values are from ordinary one-way ANOVA with Tukey’s multiple comparisons test. Overexpression of alternative alleles of *ERAP1* was confirmed by western blot. (M) Model of how rs27895 affects *ERAP1*’s transcript and protein and the resulting effect on viral burden. The reference allele of rs27895 encodes cytosine on the genome reference strand and guanine on the transcribed strand. This reference allele encodes glycine at position 346 of ERAP1 and is associated with reduced viral burden in cells. The alternate allele of rs27895 encodes thymine on the genome reference strand and adenine on the transcribed strand. This alternate allele encodes aspartate at position 346 of ERAP1 and is associated with increased viral burden in cells

Examination of stratified quantile-quantile (QQ) plots,^38^ restricted to eQTLs (2-step FDR < 0.05 in uninfected or IAV infected; minor-allele frequency [MAF] > 0.1), demonstrated deviation from neutral expectation (Figure 3A). rs12103519, associated with expression of *tumor necrosis factor superfamily member 12* (*TNFSF12*; uninfected RASQUAL 2-step FDR = 0.001, IAV RASQUAL 2-step FDR = 0.02) had the strongest association with mean IAV reads (p = 1.3 × 10^−5^). Similarly, stratification of nonsynonymous variants also demonstrated deviation from neutral expectation (Figure 3B), with a missense SNP (rs27895; G346D) in *ER aminopeptidase 1* (*ERAP1*) having the lowest p value (p = 2.9 × 10^−5^; Figures 3C and S3C). We tested both *TNFSF12* and *ERAP1* for a role in regulating IAV infection by reducing expression with small interfering RNA (siRNA) and assessing infection using an IAV mNeon reporter strain.^39^ While knockdown of *TNFSF12* had no effect (Figure 3D), *ERAP1* siRNA in LCLs led to a consistent decrease in IAV infection, suggesting a proviral role (Figure 3E; p = 0.01 in NA19020, p = 0.01 in NA19399). We used the same mNeon reporter to test whether the association of rs27895 with mean viral reads could be recapitulated using the percentage of mNeon+ cells. Across individual infections of 48 LCLs (the same LCLs as scHH-LGC), we observed a significant association of rs27895 with the percentage of mNeon+ cells having the same direction of effect (Figure S3D; p = 0.04).

*ERAP1* encodes an aminopeptidase, with the canonical function of trimming N-terminal residues from peptides for major histocompatibility complex (MHC) class I presentation.^40–42^ The derived T allele of rs27895 is associated with higher mean IAV reads and encodes a G346D amino acid change. Notably, both *ERAP1* and the related *ERAP2* have been reported to undergo IAV-mediated changes in expression, but the functional consequences of *ERAP1* induction^43^ and *ERAP2* alternative isoform usage^44^ on IAV infection are unknown. Importantly, rs27895 is not in linkage disequilibrium (LD) with the *ERAP2* SNP associated with *ERAP2* isoform usage (rs2248374^44^): R^2^ = 0.003 in European (EUR) populations and R^2^ = 0.06 in African (AFR) populations from 1000 Genomes. To confirm that *ERAP1* is proviral, we treated the A549 lung epithelial cell line with an ERAP1 inhibitor (4-methoxy-3-(N-(2-(piperidin-1-yl)-5-(trifluoromethyl)phenyl) sulfamoyl)benzoic acid^45^) and observed a dose-dependent reduction in the percentage of IAV-infected cells (Figure 3F). Notably, this ERAP1 inhibitor is highly selective for ERAP1, with a half-maximal inhibitory concentration (IC50) of 5.3 μM for ERAP1 versus >200 μM for ERAP2.^45^

While G346D is predicted by SIFT^46^ to be “tolerated” and by PolyPhen^47^ to be “benign,” this glycine residue lies within the substrate-binding pocket and, in the presence of a peptidomimic inhibitor, interacts with the first phenylalanine (F6) side chain of this ligand^48^ (Figures 3G and 3H). Moreover, residue 346 is proximal to the ERAP1 active site, which is composed of a catalytic zinc that is coordinated by residues H353, H357, and E376, the proton acceptor residue E354, and “sink” residue Tyr438.^48–50^ On the basis of our *in silico* mutational analysis of the structure of the ERAP1-inhibitor peptide complex,^48^ this SNP encoding a glycine to aspartate substitution would clearly disrupt the substrate-binding pocket of ERAP1 (Figure 3I). Indeed, the carboxylate side chain of an aspartate at ERAP1 residue 346 would result in steric clash with the side chain of any substrate other than those that have a glycine at position 6, including alanine, unless a different route to the active site is taken (Figures 3J and 3K). Consequently, there would be a significant impact on catalysis or substrate recognition or both.

Therefore, we tested the effects of overexpression of alternative alleles of *ERAP1* in A549 lung epithelial cells. Overexpression specifically of the derived T allele (aspartate; associated with higher IAV burden in scHi-HOST) caused moderately higher IAV burden (p = 0.006; 14% relative increase compared to vector), while overexpression of the ancestral C allele (glycine) had no effect (p = 0.7) (Figure 3L). These data confirm the proviral role indicated by the RNAi and inhibitor results and further demonstrates the functional effect of the G346D mutation (Figure 3M).

### Human IAV challenge supports the importance of rs27895

**Figure 4.**
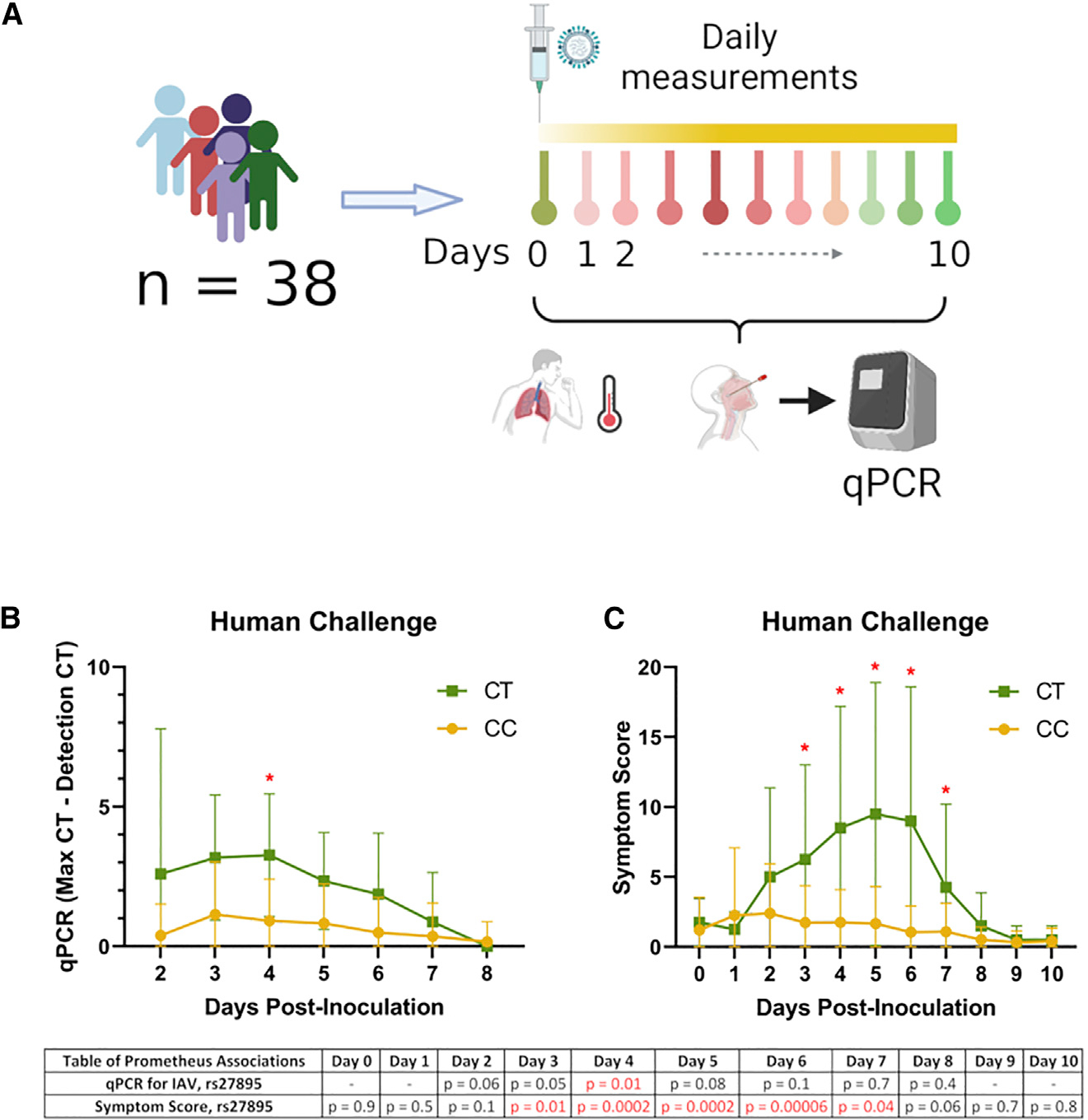
A nonsynonymous variant in *ERAP1* regulates IAV burden and symptomology in human challenge (A) Flow chart of Prometheus study. (B) rs27895 T allele is associated with IAV burden at day 4 of the Prometheus study. Viral burden was measured by nasal lavage by qPCR using pan IAV M gene primers (see STAR Methods). p values from EMMAX at each time point for rs27895 are listed below the plot. Mean and SD are plotted with lines connecting means. (C) rs27895 T allele is associated with greater symptomology at days 3–7. Jackson symptomology score was assessed daily. p values from EMMAX at each time point for rs27895 are listed below the plot. Mean and SD are plotted with lines connecting means

To determine whether rs27895 was also relevant in humans infected with IAV, we turned to a human IAV challenge study. In the Prometheus study,^51^ volunteers aged 18–55 years were enrolled if their baseline antibody titers to the CA09 (influenza A/California/04/09 (H1N1)) strain by hemagglutination inhibition assay were ≤1:10, they were healthy with no co-morbidities or risk factors for severe influenza, and they had no evidence of recent respiratory infection or significant smoking history. Thirty-eight volunteers were inoculated intranasally with CA09 IAV and underwent daily assessment and sampling by nasal lavage during the subsequent 10-day quarantine period (Figure 4A; Table S4). Both viral burden and symptoms increased over time among individuals with one copy of the T allele relative to individuals who were homozygous for the C allele. This was confirmed with association testing using EMMAX, controlling for sex, dose, and relatedness of individuals: the T allele of rs27895 was associated with higher IAV burden at day 4 (p = 0.01; Figure 4B; Table S4) and more severe symptoms from days 3 to 7, with the most significant association at day 6 (p = 6 × 10^−5^; Figure 4C; Table S4). These results demonstrate that the association of the rs27895 T allele with higher IAV burden in scHi-HOST is also observed in nasal lavage following experimental human IAV infection, where the T allele correlates with higher viral burden and more severe clinical disease.

We also examined whether the lead eQTL variant (rs12103519; Figure S4A), which was associated with expression of *TNFSF12* (Figures S4B and S4C) in scHi-HOST, showed an association in the Prometheus dataset. We observed no significant association with viral burden in human challenge (Figure S4D). Though we observed a modest association with symptoms on days 2 and 3, the effect was inconsistent with measurements at other time points, and the direction of the effect was opposite of what was predicted based on the scHi-HOST association (Figure S4E). Thus, we currently have little evidence from RNAi or human challenge to corroborate the association of rs12103519 with IAV burden in scHi-HOST, and further studies are needed to clarify the role of this and other eQTLs in IAV infection.

### Evidence that rs27895 contributes to population differentiation of IAV resistance

IAV has been responsible for at least four pandemics since the start of the 20^th^ century. Extending further back in history reveals convincing evidence of IAV pandemics since at least the 16^th^ century.^52^ Thus, IAV has had repeated impacts on human populations, consistent with pathogens serving as strong agents of natural selection.^53–55^

**Figure 5.**
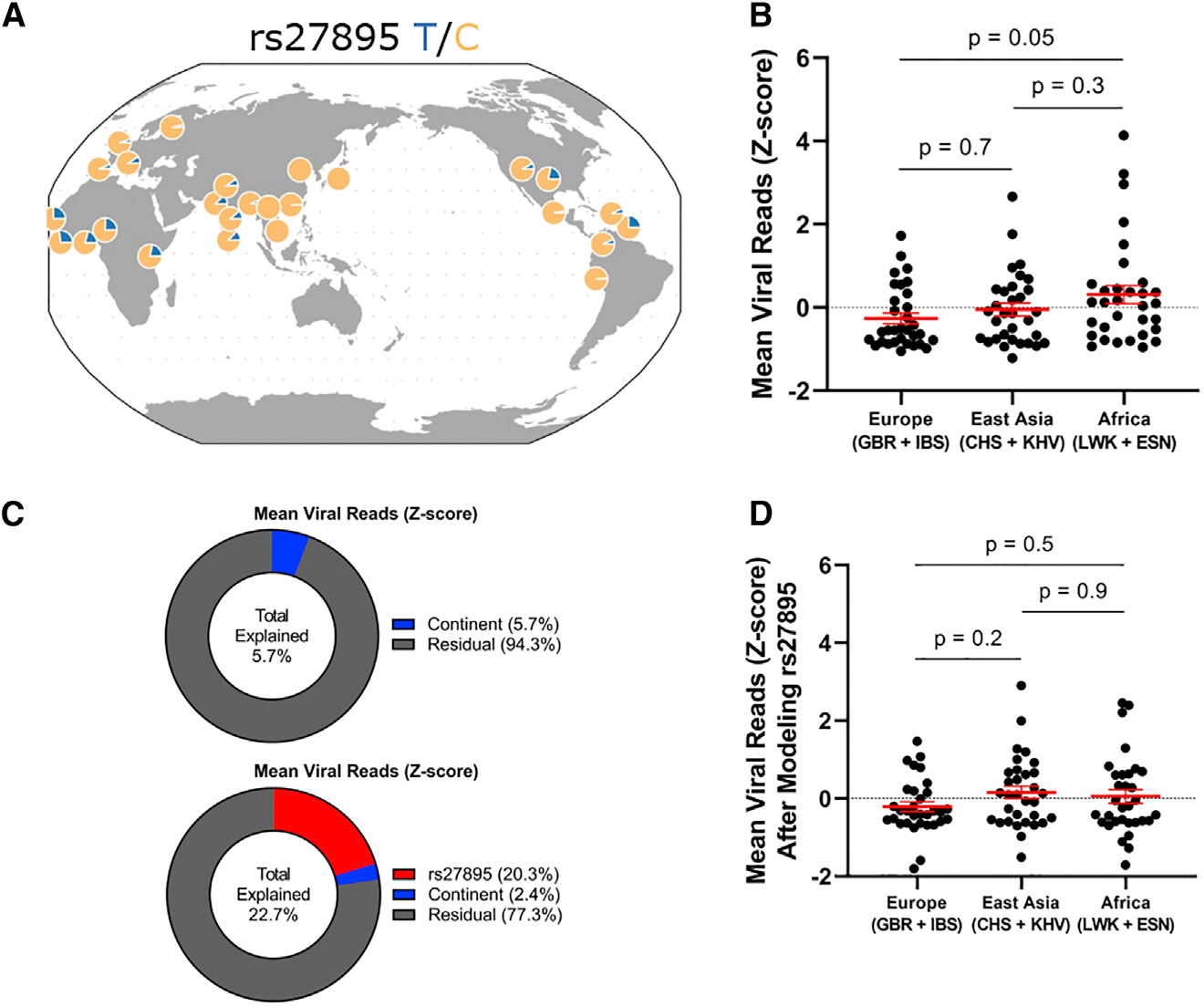
Evidence that population differentiation of rs27895 contributes to genetic resistance to IAV in European and East Asian populations (A) Geographic distribution of rs27895 is consistent with positive selection of the ancestral C allele in East Asian and European populations. (B) Combined analysis of scHH-LGC and scHH-EIK shows LCLs derived from two European and two East Asian populations are resistant to IAV compared with LCLs from two African populations. p value from ordinary one-way ANOVA with Tukey’s multiple comparisons test. This trend was also observed using the non-parametric Kruskal-Wallis test but did not reach statistical significance (p = 0.1). (C) Linear modeling of the effects of rs27895 and continent on mean IAV burden in LCLs suggests that rs27895 contributes to the population differentiation of this phenotype. (Top) Percentage explained for continent alone was modeled by lm(mean viral reads ~ continent) in R. (Bottom) Percentage explained for bivariate analysis was modeled by R function lm(mean viral reads ~ rs27895 + continent), with continent being modeled on the residual of mean viral reads after regressing out the effect of rs27895. (D) Residual phenotypic variation after removing the effect of rs27895 confirms decrease in total continental effect consistent with known allele frequency differences between populations. p value from ordinary one-way ANOVA with Tukey’s multiple comparisons test

The age and geographic pattern of rs27895 support a model where this SNP was present prior to the out-of-Africa expansion, spread throughout the world, but the resistant, ancestral C allele was selected for by IAV or other pathogens, particularly in East Asian populations. First, rs27895 has an ancient origin based on the Genealogical Estimation of Variant Age approach and resource^56^ (Figure S5A). These data indicate that the SNP is more than 500,000 years old, well before the earliest dispersals of *Homo sapiens* out of Africa around 210,000 years ago^57^ and consistent with selection at this locus acting on standing variation. Second, the derived T allele of rs27895 is the minor allele throughout the world (MAF = 10% in 1000 Genomes) and is most common in AFR populations (up to 27% in Mende in Sierra Leonne) (Figure 5A; map generated from Marcus and Novembre^58^). In contrast, the ancestral C allele, associated with IAV resistance, is most common in Asia and has become nearly fixed in East Asian populations (out of 1,008 rs27895 alleles, only one is T in Chinese Dai in Xishuangbanna, China [CDX]; Han Chinese in Beijing, China [CHB]; Southern Han Chinese [CHS]; Japanese in Tokyo, Japan [JPT]; and Kinh in Ho Chi Minh City, Vietnam [KHV] 1000 Genomes populations). This is confirmed in gno-mAD,^59^ where only 15 T alleles were detected out of 19,952 East Asian alleles. Notably, for the 10 known IAV pandemics prior to the 2009 swine flu pandemic, all were likely to have originated in Asia.^60^ We speculate that prior to the extensive human migrations that facilitate spread during pandemics, IAV outbreaks in Asian populations may have acted as a naturally selective force to increase the frequency of resistance alleles such as the rs27895C allele.

If the rs27895C allele was selected via pressures from IAV, one would predict greater IAV resistance in regions where the C allele is prevalent. Indeed, we find that LCLs from populations with higher rates of the C allele are overall protected from IAV *in vitro*. A combined dataset of scHH-LGC and scHH-EIK demonstrated lower mean viral reads in LCLs from EUR populations (GBR + IBS; 91% C allele) and East Asian populations (CHS + KHV; 98% C allele) compared with AFR populations (LWK + ESN; 75% C allele) (Figure 5B), though the differences within a continental group were much larger than differences between continental groups (Figure 5C, top, continent effect 5.7% of total variance). Linear regression of the effect of rs27895 and the continental group on IAV burden demonstrated that the amount of variation explained by the continental group is reduced if rs27895 is incorporated into the model (Figure 5C, bottom, rs27895 effect 20.3% total variance, continental effect 2.4% of residual variance). Specifically, removing the effect of rs27895 reduces IAV burden in the AFR continental group (mean from 0.31 to 0.05), consistent with the higher frequency of the susceptible derived allele in Africa. Additionally, removing the effect of rs27895 increased residual burden in the EUR continental group (mean from −0.26 to −0.21) and the East Asian continental group (mean from −0.05 to 0.16), consistent with the low frequency of the susceptible derived allele in Europe and the even lower frequency in East Asia (Figure 5D, compare with Figure 5B). The continental differences were also observed when the 48 scHH-LGC LCLs were infected and assayed individually with mNeon IAV (Figure S5B), though statistical significance was not reached in this smaller dataset. Ultimately, this observation awaits replication in an independent dataset.

## DISCUSSION

Here, we have developed a rapid scRNA-seq method to identify genetic susceptibility to cellular infection. Once an infection assay has been optimized for a particular pathogen, scHi-HOST can be carried out and analyzed within a few weeks. In the case of IAV, LCLs are highly infectable, consistent with B cells being direct targets for IAV entry,^20–22^ proliferating in response to IAV,^61–63^ and eventually undergoing cell death,^22,64^ likely contributing to lymphopenia during severe infection.^65^ Additionally, IAV infection induces global transcriptional changes in B cells, many mediated by IFN.^66,67^ As new IAV threats emerge from animal reservoirs, testing these new reassorted strains with scHi-HOST could serve as an early warning of pandemic potential as well as common genetic resistance to these strains in human populations.

scHi-HOST can be increased in scale for greater power. Our proof of concept using 96 LCLs allowed for hundreds of individual cells to be assayed for each LCL but required narrowing the genetic search space to nonsynonymous variants with high MAFs to reveal the SNP in *ERAP1* that was validated experimentally and in human challenge volunteers. Notably, the genome-wide significant hit identified by scHi-HOST (rs1923314) does show an association with the COVID19hg A1 phenotype (very severe respiratory confirmed COVID versus not hospitalized COVID; p = 0.0004; COVID-19 HGI data freeze 4^68^) with directionality consistent with scHi-HOST (A allele associated with severe COVID and greater mean IAV burden), but further work is necessary to validate the relevance of this SNP to viral infection. As scRNA-seq throughput and cost continue to improve, we anticipate increasing the cells in a single scHi-HOST experiment by an order of magnitude or more, likely revealing high-confidence genome-wide significant hits for further validation experimentally and in human challenge datasets.

scHi-HOST allows for identification of SNPs whose effects on gene expression help dictate the outcome of cellular host-pathogen interactions. Most eQTLs and even response eQTLs did not contribute to control of IAV burden in LCLs. This is a similar challenge to immunologists who confront the fact that ISGs comprise perhaps 10% of the genome but that, individually, most have little effect against an individual infection.^69,70^ Regardless, our finding that high expression of IFNs and ISGs across LCLs prior to infection is correlated with lower IAV burden confirms the importance of these genes in aggregate and warrants additional experimental dissection. However, our identification and experimental validation of a nonsynonymous variant in *ERAP1* as a major regulator of IAV in cells and humans underscores that despite noncoding variants receiving much attention as accounting for most of the identified GWAS risk alleles of common diseases,^71^ coding variation should not be ignored when trying to understand human genetic susceptibility to infectious diseases.

How ERAP1 serves as a proviral factor in IAV infection is unknown. The canonical function of ERAP1 is to trim peptides to a preferred 9-residue length for MHC class I loading. MHC class I is then trafficked from the endoplasmic reticulum (ER) to the cell surface for surveillance by T cell receptors on cytotoxic T cells.^72^ This has been reported to be an IAV-regulated process: infection with H1N1 activates p53 to increase *ERAP1* expression, resulting in increased MHC class I presentation.^43^ Our data indicate that ERAP1’s role may be complex, with our association and functional data demonstrating a proviral role for ERAP1, dependent on the rs27895 G346D polymorphism. Several other ERAP1 missense variants have been associated with inflammatory diseases, including rs30187 (K528R) with ankylosing spondylitis (AS; p = 4 × 10^−45;73^) and rs27044 (Q730E) with psoriasis (p = 8 × 10^−21;74^). These SNPs do not show an association with mean IAV burden in scHi-HOST (p = 0.73 and p = 0.57). Conversely, rs27895 is not associated with AS (p = 0.83 from downloaded summary statistics from EBI-GWAS catalog^73^) or psoriasis (p = 0.40 from downloaded summary statistics from EBI-GWAS catalog^75^). This specificity highlights that different ERAP1 variants have different roles in diseases that may be due to changes in the MHC class I antigen repertoire. How changing the composition of the MHC class I antigen repertoire (or the peptide composition within the ER lumen) could impact IAV infection is unclear. However, we speculate that as far as IAV infection of single cells is concerned, changes in the peptidome could be less important than their indirect effects on the amount of sialic acid (the ligand for IAV hemagglutinin [HA] protein) at the cell surface: as the amount of MHC class I found on the plasma membrane is regulated by the level of expression of *ERAP1*,^43^ and as MHC class I accounts for up to 26% of the α2,6-sialic acid at the cell surface,^76^ rs27895 may be indirectly dictating the amount of sialic acid available to bind IAV to facilitate entry.

Alternatively to affecting MHC class I presentation, ERAP1 has functions beyond MHC class I peptide trimming that may contribute to its role in IAV infection. This includes regulation of immune cell activation and cytokine production through secreted ERAP1.^77,78^ However, scHi-HOST is a pooled infection assay and thus would not be expected to identify genetic differences that impact secreted factors acting through non-cell-autonomous effects. ERAP1 also regulates ectodomain shedding of cell-surface receptors.^79,80^ Thus, while our work demonstrates the importance of rs27895 on IAV infection in both cells and humans, future studies will decipher how ERAP1 D346 specifically exerts its proviral function.

While the geographic distribution of rs27895 considering the history of IAV pandemic origins and our association and functional data suggests that variation in *ERAP1* may have helped humans adapt to IAV infection, human evolution of complex cellular phenotypes of infection are undoubtedly polygenic. Our data with LCLs showing IAV resistance in broadly EUR and East Asian populations contrast with recent data using monocytes and peripheral blood mononuclear cells (PBMCs) that shows resistance in AFR populations.^30,81^ What may be critical is the timing of measurement, with these studies using an early 6-h time point compared with our 24-h timepoint. Indeed, Randolph et al. show that greater EUR ancestry is associated with higher IFN levels and higher IAV burden at 6 h, but that by 24 h, PBMCs with a higher IFN response had substantially lower IAV burden.^81^ This is reminiscent of our finding that IFNα levels prior to infection are correlated with lower burden by 24 h (see Figure 1K). Differences in cell type may also play a role, as Randolph et al. demonstrate many cell-type-specific ancestry effects on IAV-induced transcriptional changes, though they also note that the ancestry effect on the IFN response is broadly conserved across all PBMC types.

Ultimately, we hope for broad adoption of scHi-HOST as a generalizable tool for rapid assessment of genetic susceptibility and resistance to pathogens that are important for human health or that pose an emerging threat. In addition to surveillance of emerging IAV strains, scHi-HOST can readily be applied to other pathogens with polyadenylated transcripts for identifying eQTLs in response to pathogen, population differences in resistance and susceptibility, and the genetic differences underlying this variation. Such genetic differences could reveal targets for drug development or FDA-approved drugs for repurposing as important prophylactics or therapeutics for future pandemics.

### Limitations of the study

The conclusions of our study are generally limited by the use of LCLs in scHi-HOST; some susceptibility alleles identified in LCLs may be cell-type specific and not predictive of risk in humans. Further, because LCLs are pooled in scHi-HOST, non-cell-autonomous effects may be obscured due to paracrine signaling between cells of different genotypes. Our allele-specific eQTL analysis is limited by the 3′ transcript bias inherent in 10x Genomics scRNA-seq. Beyond our cellular screening platform, our human challenge findings are limited mainly by sample size and sampling bias. Specifically, the Prometheus study only included 38 individuals, with 0 individuals homozygous for the rs27895 risk allele.

## STAR⋆METHODS

Detailed methods are provided in the online version of this paper and include the following:

### RESOURCE AVAILABILITY

#### Lead contact

Further information and requests for resources and reagents should be directed to and will be fulfilled by the lead contact, Dennis C. Ko, M.D., Ph.D. (dennis.ko@duke.edu).

#### Materials availability

- mNeon-tagged Influenza virus strain (A/Puerto Rico/8/1934) present in this work is available upon request.
- ERAP1-rs27895(C) and ERAP1-rs27895(T) allele overexpressing A549 cell lines are available upon request.

#### Data and code availability

- Individual LCL genotyping data is available through 1000 Genomes (FTP: http://ftp.1000genomes.ebi.ac.uk/vol1/ftp/).
- Single-cell RNA-seq data have been deposited at GEO and are publicly available as of the date of publication (GEO: GSE205796).
- RASQUAL and EMMAX results are available for download at the Duke Research Data Repository (DOI: 10.7924/r4g163n0s). Any additional information required to reanalyze the data reported in this paper is available from the lead contact upon request.
- All the software packages used in this work are included and referenced in the manuscript.
- This paper does not report original code.
- Any additional information required to reanalyze the data reported in this paper is available from the lead contact upon request.

### EXPERIMENTAL MODEL AND SUBJECT DETAILS

#### Lymphoblastoid cell lines

1000 Genomes LCLs from LWK (Luhya in Webuye, Kenya), ESN (Esan in Nigeria), GBR (British in England and Scotland), IBS (Iberian in Spain), CHS (Han Chinese South), and KHV (Kinh in Ho Chi Minh City, Vietnam) populations were purchased from the Coriell Institute. LCLs were selected to achieve equal numbers of male and female in each population. LCLs were maintained at 37°C in a 5% CO2 atmosphere and were grown in RPMI 1640 media (Invitrogen) supplemented with 10% fetal bovine serum (FBS), 2 mM glutamine, 100 U/mL penicillin-G, and 100 mg/mL streptomycin.

#### A549 cell lines

A549 cells (ATCC) were grown in DMEM +10% FBS +1% Pen-Strep. ERAP1 overexpression A549 cell lines were grown in DMEM +5% FBS +1% Pen-Strep + 1% Puromycin.

#### Human challenge volunteers

The Prometheus human infection challenge study of healthy adult volunteers with Influenza A/California/04/09 (H1N1-2009) was previously described.^51^ This study was performed at Imperial College London (London, UK) in accordance with the protocol, the Consensus ethical principles derived from international guidelines including the Declaration of Helsinki and Council for International Organizations of Medical Sciences (CIOMS) International Ethical Guidelines, applicable ICH Good Clinical Practice guidelines, and applicable laws and regulations. IAV challenge study was reviewed and approved by the Institutional Review Board at Duke University and the UK Health Research Authority London-Fulham Research Ethics Committee (ref. 17/LO/0965). Written informed consent was obtained from all participants, who provided informed consent for all study procedures including genetics, before screening and enrollment. Each volunteer was pre-screened for serum antibodies against the CA09 strain by hemagglutination inhibition, followed by screening of risk factors for severe disease and at-risk close contacts. Individuals were enrolled if H1 antibody titers against the inoculating influenza strain were ≤ 1:10. Exclusion criteria included current pregnancy, chronic lung disease, smoking, bronchodilator or steroid use (last 12 months), allergic symptoms or medication for allergic symptoms (last 6 months), acute respiratory infection (last 6 weeks), or immunodeficiency. All participants were screened for further exclusion due to drug use, pregnancy, and allergic reactions that would preclude vaccine use. Screening tests included a panel of routine blood tests, tests for immune deficiency and blood-borne virus serology.

### METHOD DETAILS

#### Viruses

The PR8 (A/Puerto Rico/8/1934) mNeon-HA virus was generated and validated as previously described.^39^ Both the wild-type and PR8 mNeon-HA viruses were amplified via injection of the respective virus into 10-day-old embryonated hen eggs (Charles River) for three days at 37°C. After incubation, allantoic fluid containing the virus was harvested, briefly spun down to remove debris, and frozen at −80°C. Viruses were then titered, as previously described, via standard plaque assay on Madin-Darby canine kidney cells (MDCKs). For the human challenge study, a live wild-type GMP-certified influenza A/California/04/2009 (H1N1) was a gift from Altimmune and produced using standard methods certified by the company.

#### Viral infection of pooled lymphoblastoid cells

48 LCLs were pooled in equal numbers and added to a 24-well plate in PBS with 0.35% BSA, 2 mM glutamine, 100 U/mL penicillin-G, and 100 mg/mL streptomycin. Cells were infected with A/Puerto Rico/8/1934 at MOI 50 or left as uninfected controls. At 3 h post infection, each well was spiked with 600 μL of RPMI 1640 media (Invitrogen) supplemented with 10% fetal bovine serum (FBS), 2 mM glutamine, 100 U/mL penicillin-G, and 100 mg/mL streptomycin. At 24 h post infection, cells from each sample were collected, spun down, and resuspended in PBS with 0.04% BSA for single-cell cDNA library preparation. LCLs from LWK, GBR, and CHS were used in scHi-HOST-LGC. LCLs from ESN, IBS, and KHV were used in scHi-HOST-EIK.

#### Single-cell RNA-seq cDNA library preparation

Cell samples were counted and checked for viability on a Guava EasyCyte HT system by 7-AAD staining before they were diluted to 1 million cells/mL with an intended capture of 10,000 cells/well. Each individual well was used to generate individually barcoded cDNA libraries using the 10x Chromium Single Cell 3′ platform version 3.1 (Pleasanton, CA) following the manufacturer’s protocol. The Chromium Controller partitions the cells into nanoliter-scale gel beads in emulsion (GEMS) within which cell-specific barcoding and oligo-dT-primed reverse-transcription occurs. For scHH-LGC, 37,013 uninfected droplets were captured across 2 Chromium wells and 32,606 IAV-exposed droplets were captured across 3 wells. For scHH-EIK, 13,675 uninfected droplets were captured in 1 Chromium well and 11,264 IAV-exposed droplets were captured in 1 Chromium well.

#### Sequencing of single-cell cDNA library

cDNA samples from scHi-HOST-LGC were dual-indexed and sequenced on one Illumina NovaSeq S4 flow cell with target depth 100,000 reads per barcoded droplet. Reads were sequenced with read 1 length of 28 base pairs (bp) and read 2 length of 150 bp. cDNA samples from scHi-HOST-EIK were single-indexed and sequenced on an Illumina HiSeq system with a target depth of 50,000 reads per barcoded droplet. Reads were sequenced with read 1 length of 150 bp and read 2 length of 150 bp. This resulted in a mean depth per cell of 58,881 reads in the uninfected scHH-LGC sample and 120,712 in the IAV-exposed scHH-LGC sample. For scHH-EIK, this resulted in a mean depth per cell of 37,815 reads in the uninfected sample and 59,803 in the IAV-exposed sample.

#### Single-cell RNA-seq alignment

Raw sequencing results were processed using the 10X Genomics CellRanger 4.0 with default parameters unless otherwise indicated. Reads from each sample were mapped to GRCh37. Reads from infected samples were also mapped to the A/Puerto Rico/8/1934 genome. For scHi-HOST-EIK, since the library was single-indexed, we used 10X Genomics Index-hopping-filter to remove index-hopped reads (https://github.com/10XGenomics/index_hopping_filter) before mapping to either human or viral genome.

#### Assignment of reads to each of 48 LCLs

To assign each barcoded read to an LCL identity, we used Demuxlet.^25^ Demuxlet takes a bam file with barcoded sample reads and a VCF containing all genotypes (obtained from the 1000 Genomes Project, phase 3 release) to computationally determine the LCL contained in each GEM reaction. Here, the bam files were taken from CellRanger output, and the VCF genotype file is the same for eQTL detection and GWAS. With barcodes assigned to each of 48 LCLs, we used Subset-Bam (10X Genomics) to subset reads from each sample alignment bam into 48 LCL-specific bam alignment files. From these LCL-specific alignment bams, we used HTSeq-Count^88^ to produce counts files for differential expression, eQTL discovery, and other transcriptomic analyses.

#### Single cell RNA-seq analysis using seurat

With the filtered and annotated expression matrix outputs of the CellRanger 4.0 pipeline, we used Seurat V4^83^ to perform single-cell analysis of PR8-infected and uninfected LCLs. Seurat data was annotated with LCL identity and normalized viral reads per droplet for analysis. Analysis on feature counts data was performed using standard log-normalization. Droplets with >20% mitochondrial reads were excluded from analysis. UMAP plots of scHiHOST-LGC uninfected and IAV samples were generated by scaling and centering features in the dataset, then reducing to 15 dimensions (as informed by Seurat:ElbowPlot). K-param nearest neighbors were calculated using Seurat:FindNeighbors with 15 dimensions. These data were projected onto 2 dimensions using Seurat:RunUMAP.

#### Differential gene expression using HTSeq-Count and Deseq2

After subsetting sample alignments to LCL-specific alignments to generate pseudo-bulk alignments per LCL, feature counting was performed using HTSeq-Count^88^ with Gencode v19 feature coordinates. All differential expression testing was performed in R using the DESeq2^82^ package. Normalization of the raw counts matrix was achieved using DESeq2’s default “Median of Ratios” method. Briefly, DESeq2 computes a pseudo-reference sample using the geometric mean for each gene. It then calculates a ratio of each sample to the computed pseudo-reference for each gene. The normalization factor (“size factor” in DESeq2) for each sample is computed by taking the median of the ratios of each gene to the pseudo-reference for each sample. Finally dividing the raw counts by the size factor yields normalized counts for further analysis in DESeq2. PCA of uninfected and infected cells was performed using variance-stabilizing transformation (vst) of the counts matrix and principal components were calculated on the 40,000 most variably expressed features.

#### Allele specific expression analysis using RASQUAL

We used RASQUAL v1.1^31^ to identify allele-specific eQTLs (aseQTLs). RASQUAL uses a probability decomposition approach to estimate aseQTLs by jointly modeling both total allele-specific count and total fragment count. RASQUAL requires two main input files, a genotype file (vcf format) including the phased genotypes and reads counts for reference allele and alternative allele for each variant for each sample, and a gene expression file including read counts of all genes.

We downloaded phased genotype data (vcf format in GRCh37) from the 1000 Genomes Project Phase 3 (available at http://ftp.1000genomes.ebi.ac.uk/vol1/ftp/), and filtered using bcftools v1.15 with parameters of “-q 0.01:minor -m 2 -M 2” to keep only biallelic SNPs and those with minor allele frequency more than 0.01. LCL-specific pseudo-bulk transcriptome alignments were obtained from Demuxlet and Subset-Bam as above. Given phased genotypes and individual sample alignment, we then quantified allele-specific read counts overlapping with biallelic variants using the ASEReadCounter function from GATK v3.8. The following parameters were used: “-U ALLOW_N_CIGAR_READS -minDepth 1 –allow_potentially_misencoded_quality_scores –minMappingQuality 10 –minBaseQuality 10”.

For gene expression input files, featureCounts^87^ was used to count reads per gene for each individual against GRCh37 reference genome with default parameters. We kept genes with >5 reads in >5 LCLs. Gene counts were then corrected using RASQUAL’s GC-correction to remove potential sample-specific GC bias. We included sex, the top 2 genotypic PCs, and transcriptome library size as covariates calculated using Plink v1.9.^89^ For each gene, SNPs within a 1 megabase window from each gene’s start and end position were included in our analysis. The final command and parameters were “tabix filtered.genotype.vcf.gz Chromosome:regionStart-regionEnd | rasqual -y expression_Y.bin -k size_factor.K.bin -n NumberSampleSize -l NumberCisSNPs -m NumberFeatureSNPs -s exon_startposition -e exon_endposition -t -f GeneName”.

Increasing the number of genotypic PCs to 5 or 10 resulted in similar results to those using the top 2 genotypic PCs. The eQTLs from incorporating the top 5 PCs captured ~93% of eQTLs from using the top 2 PCs, while incorporating the top 10 PCs captured 87% of eQTLs observed using the top 2 PCs (Figure S2D). Comparisons of eQTL effect sizes show high consistency, with minimum correlation of R > 0.97 (p < 2.2 × 10^−16^ for all R values in Figure S2E). For the 3 eQTLs specifically mentioned in the manuscript, the p values are only modestly different (Figure S2F). We did note rare instances where genes, particularly in the HLA region, demonstrated a bimodal distribution of expression, which likely resulted in false positive associations. We also observed instances of low p values being driven by allelic imbalance of a small number of heterozygous individuals, which changed direction with incorporation of additional genotypic PCs. Therefore, we strongly encourage users of scHi-HOST datasets to plot and carefully examine genotypic median and allelic imbalance plots in evaluating whether specific eQTLs are worth further characterization. However, overall, the eQTL mapping is largely consistent whether 2, 5, or 10 genotypic PCs are included.

RASQUAL’s permutation function, “–random-permutation”, was used to randomly shuffle the sample labels of each feature/gene, such that gene expression counts were assigned to random individuals. After the permutations, association is calculated between genotypes and gene expression using the RASQUAL model to generate the empirical null distribution. This permutated distribution showed minimal deviation from the theoretical null.

The nominal p values of all variants within each gene were corrected using the Benjamini-Hochberg method, and significant aseQTLs for each gene was defined using a 5% false discovery rate (FDR).

#### Visualization of ASE using phASER-POP

We used phASER-POP to aid in the plotting of allele-specific expression data. PhASER-POP implements phASER^90^ to calculate gene-level haplotypic expression per individual then combines that data across individuals to calculate the allelic fold change^35^ across the population. The tool outputs allelic fold changes for heterozygous individuals, allowing for effect size and direction of *cis*-regulatory variants to be plotted. We used our LCL-specific alignment files described above aligned to HG19 with minimum coverage of 4 allelic reads per individual per feature to calculate allelic fold changes.

#### Viral burden phenotyping

With human and viral reads assigned to each droplet, we calculated viral burden for each droplet as a function of the total reads mapped to that droplet to control for differences in read depth between droplets, resulting in normalized counts of viral reads per droplet. To get burden per LCL phenotypes, we used Demuxlet assignments of droplets to LCLs to create a distribution of viral burden for each LCL. We used the mean of each distribution as the phenotype for GWAS.

#### GWAS of viral burden

GWAS analysis on viral burden as calculated above was performed using a linear mixed model implemented in EMMAX^37^ with default parameters, a kinship matrix to control for relatedness, and sex as a covariate. The input genotypes for each LCL were obtained from the 1000 Genomes Project described as above. EMMAX controls for population stratification and cryptic genetic relatedness by incorporating a kinship matrix. The “emmax-kin” function was used to calculate the pairwise genetic relationships among individuals with Balding-Nichols algorithm. We also incorporated sex to control potential sex influence on phenotypes. In this analysis, we only carried out analysis for SNPs with minor allele frequency >10%. Furthermore, we excluded all SNPs from analysis significantly deviating from Hardy-Weinberg equilibrium (p < 10^−4^) in any of the 6 populations to remove variants that may have been affected by genotyping error. After filtering for minor allele frequency and Hardy-Weinberg equilibrium we were left with 5.2 million SNPs for GWAS. p-values reported are corrected for genomic inflation factor (*λ* = 1.05).

#### Design considerations for scHi-HOST

In designing scHi-HOST experiments, there are two components to consider: eQTLs detection and genome-wide association for viral burden. We analyzed parameters along both components.

##### 1) eQTL detection:

The combined scHH-LGC + scHH-EIK dataset consisted of sc-RNAseq from 96 pooled LCLs, with reads of single cells from the same LCL merged to conduct pseudo-bulk RNAseq and eQTL analysis. To evaluate the correlation of read depth and probability of detecting an eQTL, we split the 19,647 tested genes into 15 bins based on gene expression counts, and defined the probability of detecting eQTLs as the fraction of genes in each bin associated with at least 1 eQTL (FDR <0.05). As demonstrated for IAV-infected LCLs, the probability of detecting eQTLs increased steadily with increasing read depth, but reached a plateau at expression of ~10,000 reads (Figure S2A). This read depth corresponds to a probability of detecting an eQTL in ~50% of genes.

Next, we investigated the effect of varying depth on probability of eQTL detection. We estimate the median read depth per gene for our combined scHi-HOST dataset is 169 normalized reads, hereafter referred to as 1x. At this 1x read depth, the probability of detecting an eQTL for a typical gene in the dataset is 27%. By reducing read depth to 0.1x, the probability is nearly cut in half (15%). However, increasing the read depth from 1x to 10x had a much smaller effect, from 27% to 31%. This suggests increasing read depth results in diminishing returns in the probability of detecting an eQTL when read depth exceeds a few hundred reads and must be weighed against the increased sequencing costs.

The level of read depth/gene can be varied by altering the number of reads per droplet or the number of droplets for each LCL. Varying the reads per droplet affects the saturation curve, where additional reads beyond 50% saturation reveal more duplicates or non-unique transcripts than unique transcripts. Based on our achieved sequencing depth of 120,712 reads per droplet, we estimate a sequencing saturation of 43% for the scHH-LGC IAV sample (Figure S2B). Using this read depth per droplet and a target of 10,000 droplets per 10X Chromium well, we obtained a median of 501 droplets per LCL using 3 wells (see Figure 1C). For the scHH-LGC IAV-infected sample, this resulted in a pseudo-bulk mean read-depth/LCL of 40,187,085.

##### 2) GWAS of viral burden:

For scHi-HOST GWAS of viral burden, the key considerations are obtaining high precision in phenotype measurement and including a sufficient number of individuals for adequate power. To determine the number of droplets of each LCL to recover in order to accurately reflect the mean viral reads phenotype, we sampled different levels of cell recovery from 1 to 1,000 droplets with replacement from the distribution of mean viral reads for our most abundant LCL (n = 639 droplets, NA19399). We performed this sampling 5,000 times to calculate the SD of the resulting mean viral reads phenotype for each quantity of recovered single cells (Figure S2C). Our results suggest that a good estimate of the true phenotype can be obtained by recovering ~200 cells for each LCL. We see diminishing returns in the estimate of the phenotype beyond 200 droplets.

The number of individuals affects the power of the GWAS component of scHi-HOST, as it would any GWAS. Researchers can determine the number of LCLs to use in their screens using a GWAS power calculator for quantitative traits.^91,92^ In our case, with 96 LCLs and testing either 5.2 million SNPs genome-wide or 15,866 nonsynonymous SNPs (mAF >0.1, HWE p > 1 × 10^−4^), we achieved power of 0.008 and 0.1, respectively, to detect an association with an effect size 0.1 (using GWAPower by^91^). Our *ERAP1* SNP (rs27895, nonsynonymous) has an effect size of 0.18, and our power to detect an SNP of this effect was 0.14 and 0.51, respectively. Thus, scHi-HOST of 96 LCLs could only detect associations of massive effect size considering genome-wide SNPs but was adequately powered to detect large-effect nonsynonymous variants associated with IAV burden.

#### Enrichment analysis of eGenes

The enrichment of scHi-HOST eGenes in GTEx and enrichment of ISGs in scHi-HOST eGenes were tested using Fisher’s exact test. Significance of enrichment was calculated using the following equation:

$$p=\frac{\left(\begin{array}{c} n_{2}\\ k \end{array}\right)\left(\begin{array}{c} N_{Total}-n_{2}\\ n_{1}-k \end{array}\right)}{\left(\begin{array}{c} N_{Total}\\ n_{1} \end{array}\right)}$$

where NTotal is the total number of genes, the n_1_ and n_2_ are the number of eGenes associated with scHi-HOST and GTEx, and k is the number of eGenes shared between scHi-HOST and GTEx.

#### Viral infection of human volunteers

The Prometheus human infection challenge study of healthy adult volunteers with Influenza A/California/04/09 (H1N1-2009) was previously described.^51^ Subjects were inoculated intranasally with live GMP-certified influenza A/California/04/2009 (Altimmune) at a mean dose of 10^6^ within 6 weeks of screening. Post-inoculation, subjects were confined to an isolation facility for eight days with daily collection of clinical and physiologic findings, modified Jackson symptomology scores,^93^ nasal lavage, and blood samples. Symptom scores were recorded up to day 10 post-inoculation and follow-up interviews and physical examinations were conducted at day 14 and 28.

#### qPCR of nasal lavage fluid

qPCR was performed on nasal lavage samples as previously described.^94^ Briefly, total RNA was isolated using the QIAamp Viral RNA kit (Qiagen) according to the manufacturer’s instructions. Reverse transcription of 13 μL of total isolated RNA was achieved using the High Capacity RNA-to-cDNA kit (Applied Biosystems) according to the manufacturer’s instructions. Quantitative RT-PCR reactions for viral load were achieved using pan-IAV M gene primers and probes (forward: GACCRATCCTGTCACCTCTGAC, reverse: AGGGCATTYTGGACAAAKCGTCTA, probe: TGCAGTCCTCGCTCACTGGGCACG) with the TaqMan Universal Master Mix II (Applied Biosystems) and 7500 Fast Real-Time PCR System (Applied Biosystems). Absolute quantification was calculated using a plasmid DNA standard curve.

#### Prometheus low-pass whole genome sequencing and imputation

Whole genome sequencing was performed on Prometheus human challenge study subjects. Total genomic DNA was extracted from buffy coat using the QIAGEN DNeasy blood kit following manufacturer’s instructions (average genomic DNA concertation from 38 volunteers was 95.58 ng/μL). Whole genome sequencing was carried out by BGI using BGI DNBSEQ low-pass genome sequencing to 4x coverage. Short reads were aligned to human genome GRCh37 and imputed through the Gencove ImputeSeq pipeline using the 1000 Genome Project Phase 3 reference panel.^95^ Imputed variants were filtered using *bcftools* v1.15,^96^ including minor allele frequency >0.05, genotype missingness <0.2, sample missing calls <0.2. In the subsequent analysis, only autosomal biallelic variants were included. EMMAX was used to run GWAS on qPCR of IAV load and symptom scores, with details described above.

#### LCL RNAi experiments

LCLs (2 × 10^5^ cells) were treated for three days in 500 μL of Accell media (Dharmacon) with either non-targeting Accell siRNA #1 or an Accell SmartPool directed against human *ERAP1* or *TNFSF12* (1 μM total siRNA; Dharmacon) in a 24-well TC-treated plate. After 3-day incubation with siRNA, cells were plated at 30,000 per well in 50μL PBS with 0.35% BSA, 2 mM glutamine, 100 U/mL penicillin-G, and 100 mg/mL streptomycin in 96-well plates. Infections were conducted at MOI 50 with PR8-mNeon-HA for 3 h before wells were spiked with 75μL RPMI (10% FBS +1% Pen-Strep). Cells were assayed for Percent mNeon + cells using a Guava Easycyte flow cytometer at 24 h post-infection. RNA was collected from 2 × 10^5^ pelleted cells using the RNeasy mini kit (Qiagen). qPCR using primers specific for *ERAP1* or *TNFSF12* were used to validate knockdown relative to 18S rRNA.

#### ERAP1 inhibitor in A549 experiments

A549 cells were plated at 25,000 cells per well in 50 μL growth media (DMEM +10% FBS +1% Pen-Strep) in 96 well plates. 1 day after plating, media was removed and replaced with 50 μL PBS with 0.35% BSA, 2 mM glutamine, 100 U/mL penicillin-G, and 100 mg/mL streptomycin and 1, 5, or 10 μM ERAP1-IN-1 (CAS: 865,273-97-8, MedChemExpress), or equal volume DMSO (vehicle). After 30-min incubation, cells were infected at MOI 1 with PR8-mNeon-HA for 1 h before infectious media was removed and replaced with post-infection media (Opti-mem + 0.01% FBS +1% Pen-Strep + 0.35% BSA). 1-day post-infection, cells were trypsinized and assayed for percent mNeon + cells using a Guava Easycyte flow cytometer.

#### Overexpression in A549

For lentivirus production, HEK-293T cells were transfected with 1 μg pLEX-ERAP1, 0.4 μg pMD2.G and 1 μg pCMVΔ8.74. Lentiviral supernatant media was collected after 72 h. Next, A549 cells in a 24-well plate were transduced with 1 mL lentivirus and selected with 1 μg/mL puromycin after 48 h. After 3 days selection, surviving cells were expanded.

Cell lines were plated at 25,000 cells per well in 50 μL selection media (DMEM +5% FBS +1% Pen-Strep + 1% Puromycin) in 96-well plates. 1 day after plating, media was removed and replaced with 50 μL PBS with 0.35% BSA, 2 mM glutamine, 100 U/mL penicillin-G, and 100 mg/mL streptomycin and infected at MOI 0.1 with PR8-mNeon-HA for 1h before infectious media was removed and replaced with selection media. 1 day post-infection, cells were trypsinized and assayed for percent mNeon + cells using a Guava Easycyte flow cytometer.

#### *In silico* mutagenesis of ERAP1 and 10-mer peptide

*In silico* mutational analysis of ERAP1 bound to the 10-mer peptide (PDB: 6RQX) was performed using the Pymol mutagenesis function (The PyMol Molecular Graphics System, Version 1.8.6.0 Schrodinger, LLC).

### QUANTIFICATION AND STATISTICAL ANALYSIS

The quantitative and statistical analyses are described in the relevant sections of the Method details or in the figure legends.

## Supplementary Material

1

2

3

4

5

## Figures and Tables

**KEY RESOURCES TABLE:** 

REAGENT or RESOURCE	SOURCE	IDENTIFIER

Virus strains		

A/Puerto Rico/8/1934	Gift from Dr. Nicholas Heaton	N/A
A/California/04/09	Gift from Altimmune	N/A

Chemicals, peptides, and recombinant proteins		

ERAP1-IN-1 ERAP1 inhibitor	MedChemExpress	CAS: 865273-97-8

**Antibodies**		
Anti-alpha Tubulin antibody	abcam	RRID:AB_2890906
Anti-ERAP1 antibody	Santa Cruz	RRID:AB_10708722
Goat anti-rabbit IgG antibody	Life Technologies	RRID:AB_2534776
Goat anti-mouse IgG antibody	Life Technologies	RRID:AB_2534745
Critical commercial assays		

10x Chromium Single Cell 3′ platform version 3.1	10X Genomics	https://www.10xgenomics.com/
QIAamp Viral RNA kit	QIAGEN	52904
QIAGEN DNeasy blood kit	QIAGEN	69504
QIAGEN RNeasy mini kit	QIAGEN	74004
High Capacity RNA-to-cDNA	Applied Biosystems	4387406
TaqMan Universal Master Mix II	Applied Biosystems	4440038

Deposited data		

scRNA-seq	this manuscript	GEO: GSE205796
Emmax and Rasqual Results	this manuscript	DOI: 10.7924/r4g163n0s

Experimental models: Cell lines		

LCLs	Coriell	https://www.coriell.org/1/NIGMS
ERAP1 Overexpression A549s	This manuscript	N/A

Oligonucleotides		

ERAP1 Accell SMARTpool siRNA	Dharmacon	E-005787-00-0010
TNFSF12 Accell SMARTpool siRNA	Dharmacon	E-010629-00-0010
ERAP1 TaqMan assay	Thermo	Hs00429970_m1
TNFSF12 TaqMan Assay	Thermo	Hs00387540_g1

Software and algorithms		

CellRanger v4.0	10X Genomics	https://support.10xgenomics.com/single-cell-gene-expression/software/pipelines/latest/what-is-cell-ranger
index-hopping filter v1.0.1	10X Genomics	https://github.com/10XGenomics/index_hopping_filter
Demuxlet	Kang et al.^25^	https://github.com/VincentGardeux/demuxlet
Subset-bam v1.1.0	10X Genomics	https://github.com/10XGenomics/subset-bam
HTSeq-Count v0.13.5	Love et al.^82^	https://htseq.readthedocs.io/en/release_0.11.1/count.html
Seurat v4	Hao et al.^83^	https://satijalab.org/seurat/
DESeq2	Butler et al.^24^	https://bioconductor.org/packages/release/bioc/html/DESeq2.html
GSEA v4.1	Subramanian et al.^28^	https://www.gsea-msigdb.org/gsea/index.jsp
Rasqual v1.1	Kumasaka et al.^31^	https://github.com/natsuhiko/rasqual
GATK v3.8	Van der Auwer and O'Connor^84^	https://gatk.broadinstitute.org/hc/en-us
EMMAX	Kang et al.^37^	https://genome.sph.umich.edu/wiki/EMMAX
Plink v1.9	Chang et al.^85^	https://www.cog-genomics.org/plink/
R v4.0	R Core Team, 2021	https://www.r-project.org/
Pymol v2.0	The PyMOL Molecular Graphics System, Version 2.0 Schrödinger, LLC.	https://pymol.org/2/#page-top
Graphpad Prism	GraphPad Software, Inc.	www.graphpad.com
Gencove ImputeSeq	Gencove	www.gencove.com
Bcftools v1.15	Li^86^	https://samtools.github.io/bcftools/bcftools.html
featureCounts v2.0	Liao et al.^87^	http://subread.sourceforge.net/

Other		

The 1000 Genomes Project	Auton et al.^19^	http://ftp.1000genomes.ebi.ac.uk/vol1/ftp/

## Highlights

- Multi-population single-cell GWAS connects SNPs with gene expression and IAV burden
- Analysis of nonsynonymous SNPs reveals ERAP1 G346D associated with viral burden
- Inhibition and overexpression of ERAP1 in A549 confirms effect on viral burden
- ERAP1 G346D is also associated with symptoms and viral burden in human challenge
