# Supplementary material for: Single-cell genome-wide association reveals that a nonsynonymous variant in ERAP1 confers increased susceptibility to influenza virus
Source: Cell Genom. 2022 Nov 9;2(11):100207. doi: 10.1016/j.xgen.2022.100207 (PMC9718543; doi:10.1016/j.xgen.2022.100207)
Supplement: Document S1. Figures S1–S5 [file mmc1.pdf]

Cell Genomics, Volume *2*

Supplemental information

Single-cell genome-wide association reveals that a nonsynonymous variant in *ERAP1* confers increased susceptibility to influenza virus

Benjamin H. Schott, Liuyang Wang, Xinyu Zhu, Alfred T. Harding, Emily R. Ko, Jeffrey S. Bourgeois, Erica J. Washington, Thomas W. Burke, Jack Anderson, Emma Bergstrom, Zoe Gardener, Suzanna Paterson, Richard G. Brennan, Christopher Chiu, Micah T. McClain, Christopher W. Woods, Simon G. Gregory, Nicholas S. Heaton, and Dennis C. Ko

Supplemental Information

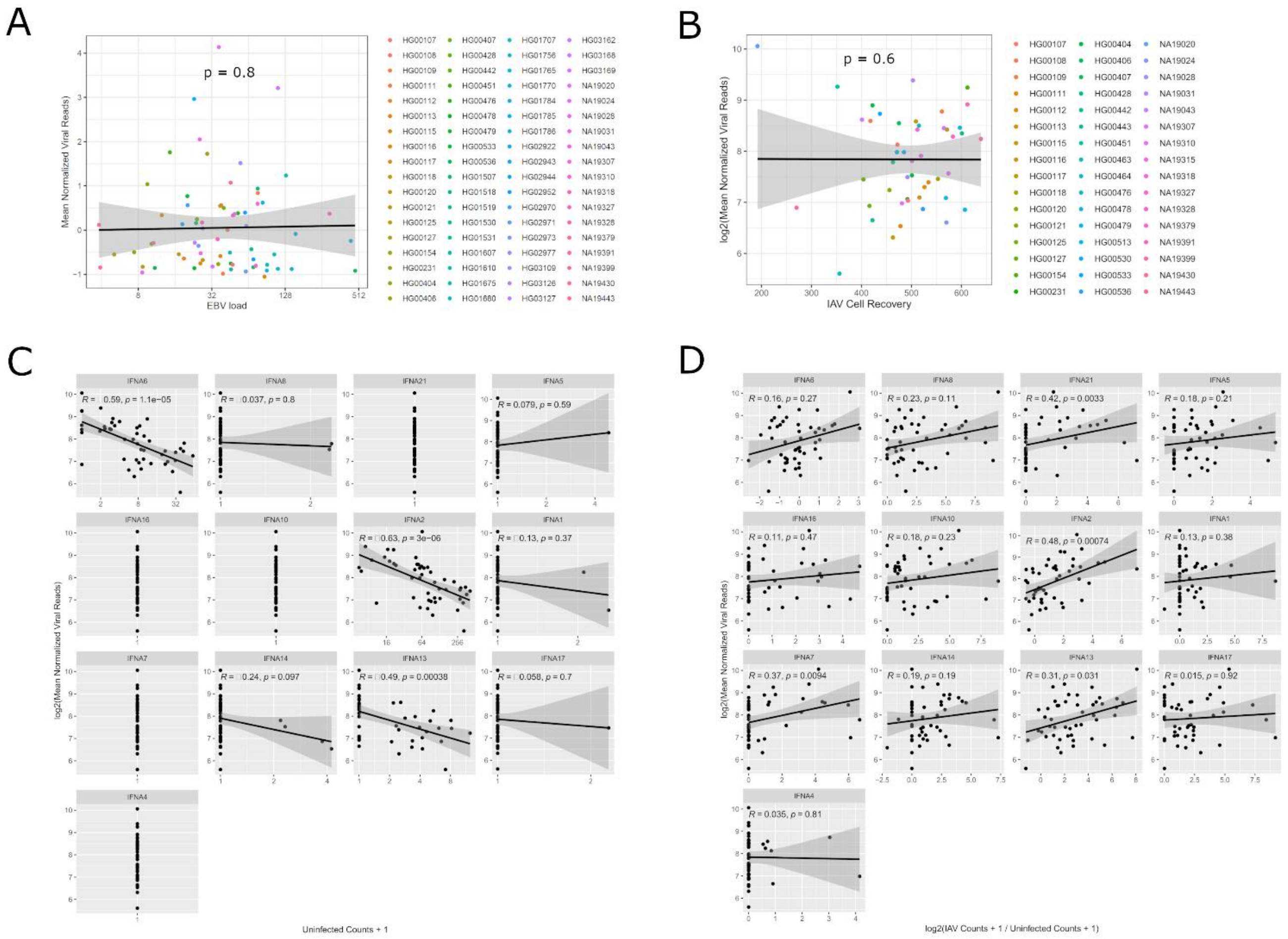

Figure S1. IFNα gene expression correlates with LCL IAV burden, but EBV burden and cell recovery do not. Related to Figure 1. (**A**) EBV copy number in LCLs does not correlate with IAV burden. EBV copy number from 72 LCLs out of 96 LCLs were obtained from (Mandage et al., 2017). A non-significant correlation (p = 0.8) was observed between EBV copy number and mean viral reads (normalized per cell) using Spearman correlation. Each point with distinct color represents an LCL. (**B**) The number of each LCL recovered for RNA-seq does not correlate with IAV burden. A non-significant correlation (p = 0.6) was observed between cell number and mean viral reads (normalized per cell) using Spearman correlation. Each point with distinct color represents an LCL. (**C**) Expression of all detected IFNα genes vs. log2(Mean Normalized Viral Reads) shows that, for expressed IFNα genes, higher basal expression is correlated with lower viral burden after infection. Correlation coefficient and p-values are from Spearman's correlation. (**D**) Induction (log2(IAV Counts + 1 / Uninfected Counts + 1) of all IFNα genes reveals that IFNα induction is correlated with higher viral burden. Correlation coefficient and p-values are from Spearman's correlation.

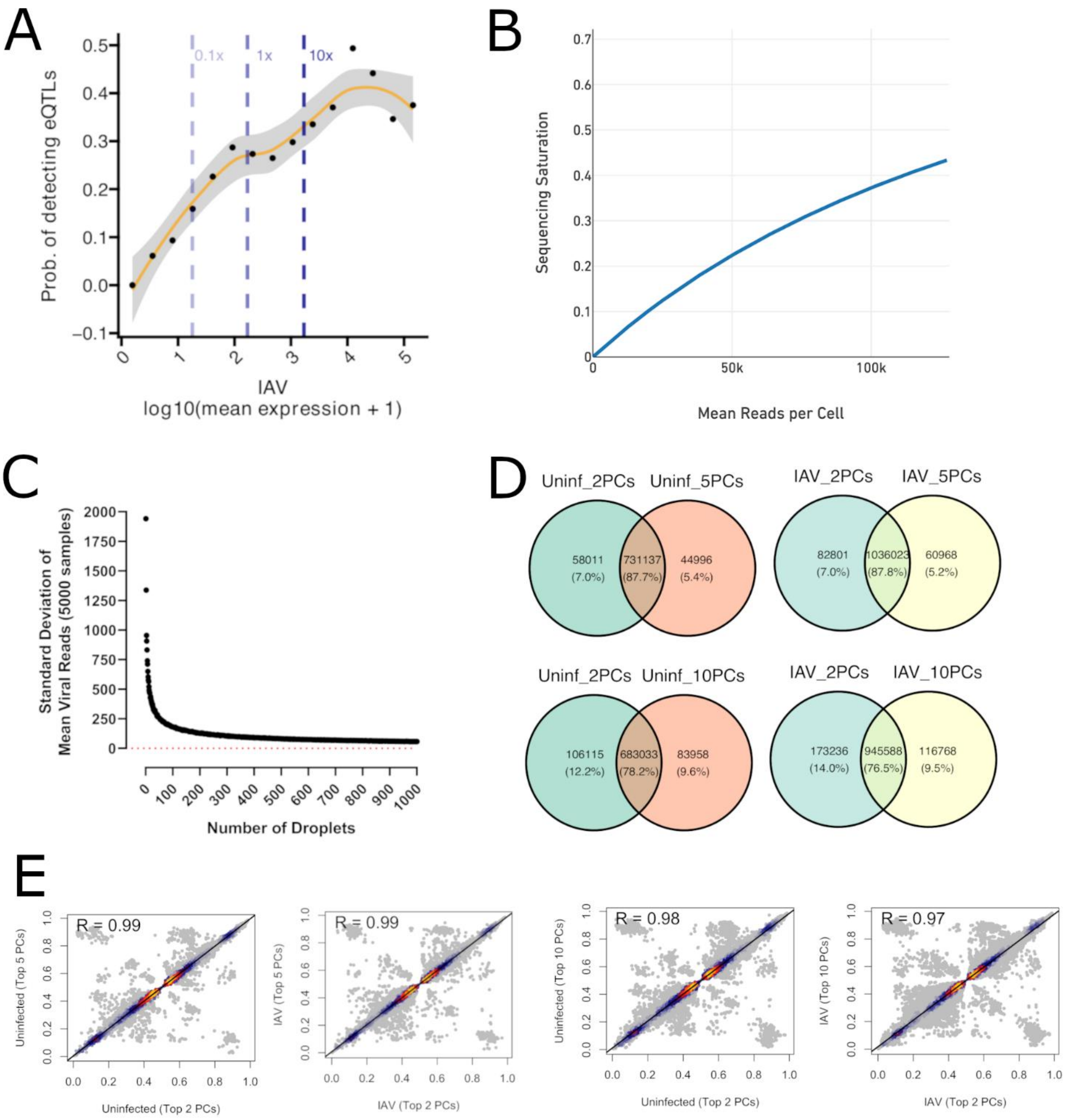

F

eQTLs	Gene	Top 2 PCs		Top 5 PCs		Top 10 PCs	
		Uninfected	IAV	Uninfected	IAV	Uninfected	IAV
rs11080327	SLFN5	2.7e-143	3.3e-253	2.0e-140	6.9e-248	2.1e-139	3.2e-246
rs10495545	NRIR	-	2.1e-05	-	4.96e-05	-	1.7e-05
rs12103519	TNFSF12	7.6e-05	2.3e-03	6.3e-03	5.6e-02	9.5e-03	-

Figure S2. Factors relevant to the design of a scHi-HOST experiment. Related to Figure 2. (**A**) Mean expression of individual genes across LCLs vs. the probability of detecting an eQTL for that gene in the IAV-

infected sample. 19647 tested genes were divided into 15 bins based on gene expression counts. The probability of detecting eQTLs was defined as the fraction of genes in each bin associated with at least 1 eQTL (FDR < 0.05). (**B**) Plot of mean reads per droplet vs. sequencing saturation for a typical 10X well from the IAV sample. (**C**) Plot of the standard deviation in the estimate of mean viral reads for each number of 1 – 1000 droplets recovered. Droplets were sampled from the distribution of mean viral reads for our most abundant LCL (n = 639), NA19399. We performed this sampling 5,000 times to calculate the standard deviation of the resulting mean viral reads phenotype for each quantity of recovered single cells. (**D**) Venn diagrams of eQTLs identified using RASQUAL (FDR < 0.05), incorporating the top 2, 5 or 10 genotypic PCs for uninfected and IAV-infected conditions. (**E**) Correlation of effect sizes of uninfected and IAV-infected eQTLs identified accounting for either the top 2 or 5 genotypic PCs (left panels) and top 2 or 10 PCs (right panels). All comparison of eQTLs were matched by rsID and Ensembl gene ID. (**F**) RASQUAL p values for 3 eQTLs described in the text, using varying numbers of top genotypic PCs. "-" indicates p > 0.05.

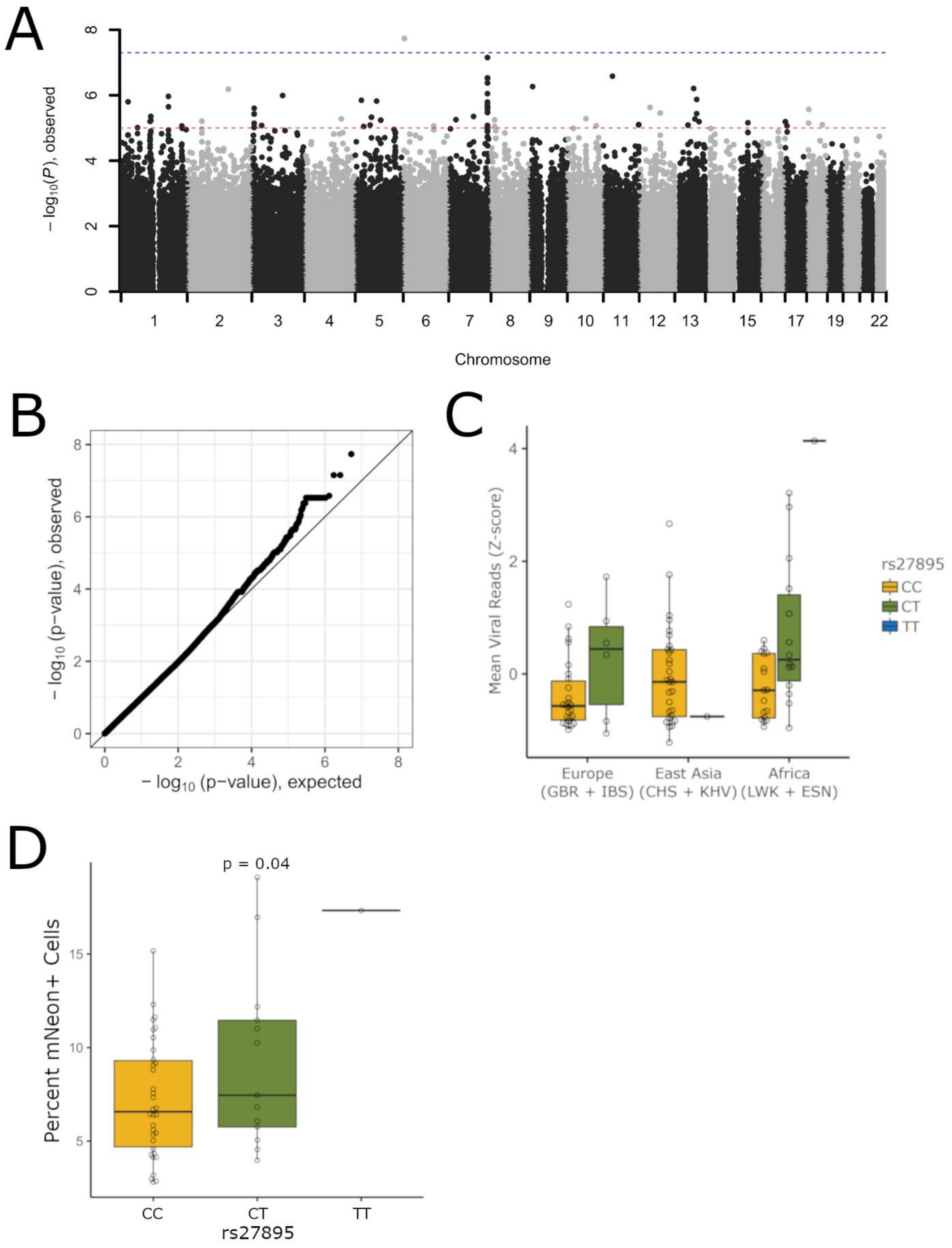

Figure S3. Cellular GWAS of mean IAV burden in LCLs. Related to Figure 3. (**A**) Manhattan plot of mean IAV burden based on p values from EMMAX using kinship matrix, sex as a covariate, and with SNPs with MAF < 10% and deviation from Hardy-Weinberg Equilibrium (p < 1x10-4) excluded. P values plotted are corrected

for genomic inflation factor (λ = 1.05). (**B**) QQ plot of the same data in A. (**C**) Plotting by continent reveals that rs27895-T is associated with increased viral reads in European and African populations while the C allele of rs27895 is nearly fixed in East Asian populations. (**D**) The same 48 LCLs used in scHH-LGC were individually assayed for the percent of mNeon-PR8 infected LCLs by flow cytometry. We observed an association with rs27895 similar to the association with the scHi-HOST mean viral reads phenotype.

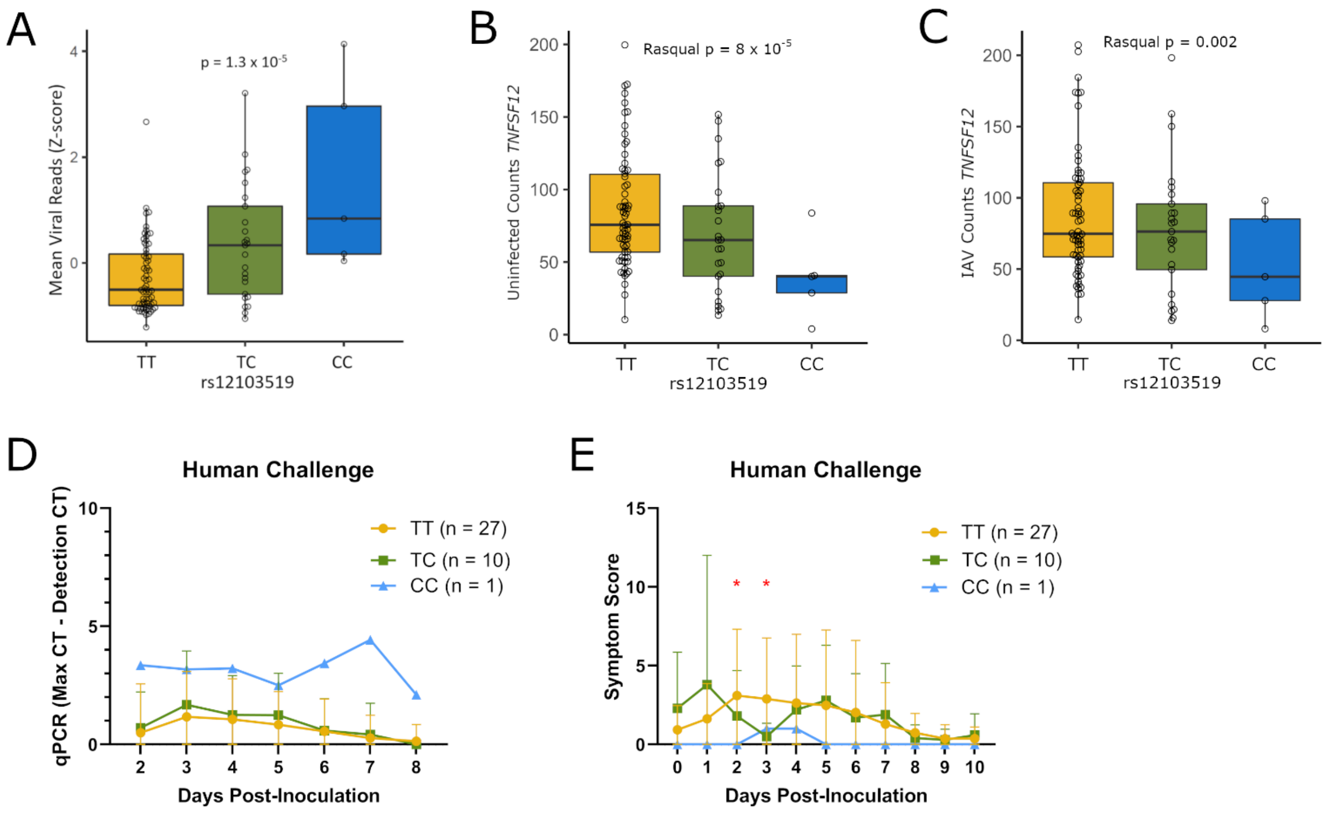

Table of Prometheus Associations	Day 0	Day 1	Day 2	Day 3	Day 4	Day 5	Day 6	Day 7	Day 8	Day 9	Day 10
qPCR for IAV, rs12103519	-	-	p = 0.7	p = 0.7	p = 0.9	p = 0.6	p = 0.5	p = 0.09	p = 0.5	-	-
Symptom Score, rs12103519	p = 0.4	p = 0.5	p = 0.05	p = 0.008	p = 0.3	p = 0.4	p = 0.3	p = 0.7	p = 0.2	p = 0.6	p = 1.0

Figure S4. rs12103519 shows an association in scHi-HOST, but not in Prometheus human challenge. Related to Figure 4. (**A**) rs12103519-C allele is associated with higher viral burden in LCLs. rs12103519-C allele is associated with reduced expression of *TNFSF12* in uninfected sample (**B**) and IAV sample (**C**). (**D**) rs12103519 is not associated with viral burden by qPCR in human challenge. (**E**) rs12103519-T allele is associated with worse symptoms on days 2 and 3 post-inoculation during human challenge. However, this effect is not consistent across other timepoints and is the opposite of the predicted direction of effect by scHiHOST (A-C).

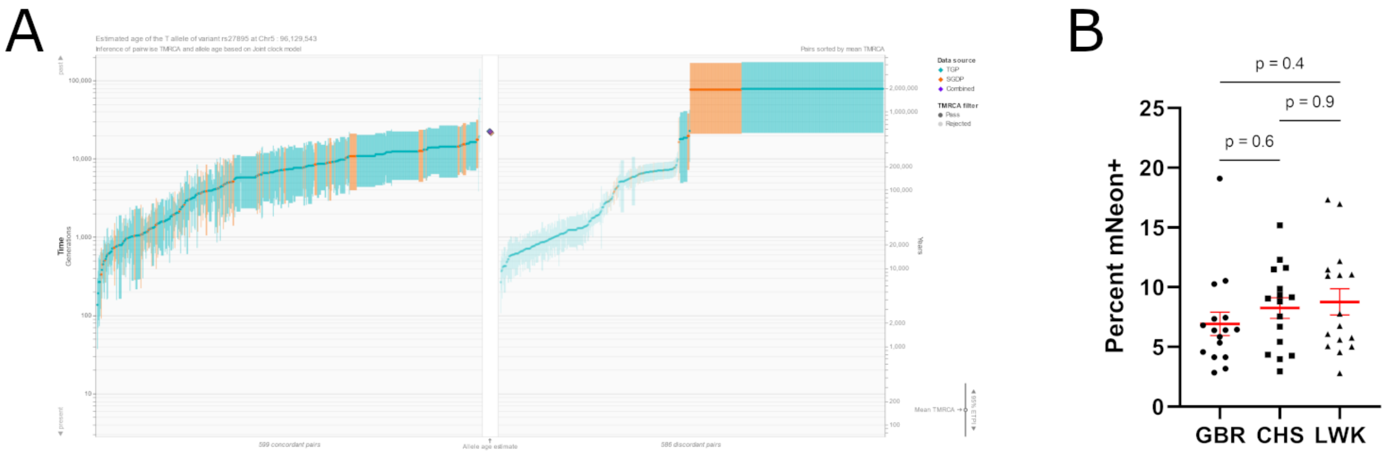

Figure S5. rs27895 was present in human populations before the out-of-Africa expansion and may explain population differences in viral burden. Related to Figure 5. (**A**) Data and figure from https://human.genome.dating suggest that_rs27895 was present in human populations before the out-of-Africa expansion. (**B**) Individual mNeon-IAV infections of the same 48 LCLs as scHH-LGC and flow cytometric measurements of the percent of infected cells. The same direction of population effect is observed as mean viral reads, LWK > CHS > GBR (see figure 5B).
